# Integrated network pharmacology and molecular modeling approach for the discovery of novel potential MAPK3 inhibitors from whole green jackfruit flour targeting obesity-linked diabetes mellitus

**DOI:** 10.1371/journal.pone.0280847

**Published:** 2023-01-30

**Authors:** Tejaswini Maradesha, Reshma Mary Martiz, Shashank M. Patil, Ashwini Prasad, Abdullatif Taha Babakr, Ekaterina Silina, Victor Stupin, Raghu Ram Achar, Ramith Ramu

**Affiliations:** 1 Department of Biotechnology and Bioinformatics, JSS Academy of Higher Education and Research, Mysuru, Karnataka, India; 2 Department of Microbiology, JSS Academy of Higher Education and Research, Mysuru, Karnataka, India; 3 Department of Medical Biochemistry, College of Medicine, Umm Al-Qura University, Makkah, Saudi Arabia; 4 Institute of Biodesign and Modeling of Complex Systems, I.M. Sechenov First Moscow State Medical University (Sechenov University), Moscow, Russia; 5 Department of Hospital Surgery 1, N.I. Pirogov Russian National Research Medical University (RNRMU), Moscow, Russia; 6 Division of Biochemistry, School of Life Sciences, JSS Academy of Higher Education and Research, Mysuru, Karnataka, India; D Y Patil Deemed To Be University: DY Patil University Deemed to be University, INDIA

## Abstract

The current study investigates the effectiveness of phytocompounds from the whole green jackfruit flour methanol extract (JME) against obesity-linked diabetes mellitus using integrated network pharmacology and molecular modeling approach. Through network pharmacology, druglikeness and pharmacokinetics, molecular docking simulations, GO analysis, molecular dynamics simulations, and binding free energy analyses, it aims to look into the mechanism of the JME phytocompounds in the amelioration of obesity-linked diabetes mellitus. There are 15 predicted genes corresponding to the 11 oral bioactive compounds of JME. The most important of these 15 genes was MAPK3. According to the network analysis, the insulin signaling pathway has been predicted to have the strongest affinity to MAPK3 protein, which was chosen as the target. With regard to the molecular docking simulation, the greatest notable binding affinity for MAPK3 was discovered to be caffeic acid (-8.0 kJ/mol), deoxysappanone B 7,3’-dimethyl ether acetate (DBDEA) (-8.2 kJ/mol), and syringic acid (-8.5 kJ/mol). All the compounds were found to be stable inside the inhibitor binding pocket of the enzyme during molecular dynamics simulation. During binding free energy calculation, all the compounds chiefly used Van der Waal’s free energy to bind with the target protein (caffeic acid: 102.296 kJ/mol, DBDEA: -104.268 kJ/mol, syringic acid: -100.171 kJ/mol). Based on these findings, it may be inferred that the reported JME phytocompounds could be used for *in vitro* and *in vivo* research, with the goal of targeting MAPK3 inhibition for the treatment of obesity-linked diabetes mellitus.

## Introduction

Obesity-linked diabetes mellitus (DM) has been considered one of the concerning health issues in the current scenario due to the uncommon surge in urbanization, sedentary lifestyle, and abnormal nutrition [1, 2]. Obesity occurs when the body stores surplus energy in the form of lipid content or fat when a person gains a high amount of energy but does not burn off the energy through exercise and physical activity. In addition to fat, hormones, cytokines, proinflammatory markers, and other substances are also increased, which are directly involved with insulin resistance [3, 4]. Therefore, in obese individuals, there is a high risk of β-islet cells of the pancreas getting damaged, resulting in the impairment of blood glucose regulation [5]. These conditions and insulin resistance elevate the risk of developing type-1 and type-2 DM. Also, the deposition of extensive lipid content in the blood vessels can lead to severe cardiac problems [2, 6, 7].

The current perspectives on the treatment of obesity-linked DM involve non-pharmacological approaches including exercise, diet control, surgery, and pharmacological approaches like insulin and non-insulin methods [7, 8]. However, the majority of the patients stick with diet control, exercise, and medicines for the control of obesity-linked DM. The disorder has no available specific therapy, except dieting and exercise. Some glucose-lowering medications are available that may promote weight loss include metformin, glucagon-like peptide-1 (GLP-1) receptor agonists, pramlintide, sodium-glucose cotransporter 2 (SGLT2) inhibitors, and dipeptidyl peptidase 4 (DPP-4) inhibitors [8]. However, the majority of these medications have been linked to adverse effects such as lactic acidosis, ketoacidosis, anemia, bone fractures, and digestive and cardiovascular issues. Because they are less toxic than synthetic medications, it is worthwhile to evaluate phytoconstituents with the potential to lower blood sugar and body fat [2, 9–11].

Different food products including milk, vegetables, and fruits have got anti-hyperlipidemic and anti-diabetic factors [14]. In the current scenario, the usage of phytochemical products for the treatment of anti-diabetic conditions has been trending, since they are associated with a low risk of developing adverse effects. Therefore, extraction of anti-diabetic components of plant products could be an essential step toward developing a better complementary alternative medicine (CAM) [12].

Traditionally, herbal preparations in the form of medicines have been used for centuries. According to The World Health Organization, more than 80% of the world population in developing countries rely primarily on plant-based medicines for basic healthcare needs [13]. Many of the *Artocarpus* species are potentially used for both medicines as well as timber in the Asian subcontinent. The most popular of the species is *Artocarpus heterophyllus* (jackfruit) belongs to the *Moraceae* family and is a common household tree in India and Bangladesh [14]. Considering its economic value and abundance, several studies have been carried out on jackfruit suggesting that it possesses diverse medicinal uses including antioxidant, anti-inflammatory, antimicrobial, anticarcinogenic, antifungal, antineoplastic, and hypoglycemic effects inhibiting melanin biosynthesis and wound healing properties [15]. The observed pharmacological properties may be attributed to the presence of various phytochemicals. Studies have also proved that the fruit contains carotenoids, flavonoids, volatile acids, sterols, and tannins which have rich antioxidant potential. Although information is available on ripe jackfruit in terms of its composition and health benefits, there is scarce knowledge on its potential in the treatment of diabetes [16].

Network pharmacology is a recently developed multidisciplinary pharmacological research area that makes use of big data and artificial intelligence [17]. It is widely used to recognize active pharmaceutical compounds and comprehend the entire mechanism of action of pharmaceuticals, providing cutting-edge technical and scientific support for novel drug research and development and clinical medication use [18]. It acts as an effective tool to screen and identify the lead therapeutic compound. In contrast to Western medicine, which is based on a single drug for a single target, phytochemical-based therapies provide their therapeutic and pharmacological effects as a whole via numerous targets and multiple components. Network pharmacology, which is based on the poly target-poly compound approach, attempts to investigate the efficacy of medications on a holistic level, shifting research methods away from the established one potential drug-one target model and towards a developing one drug-network targets mode [19, 20]. Hence, we aim to investigate the pharmacological activities of the phytocompounds from *A*. *heterophyllus* against obesity-linked DM through network pharmacology and molecular modeling methods.

## Materials and methods

### Construction of phytocompounds library

A total of 120 compounds were identified from whole green jackfruit flour methanol extract (JME) **(S1–S4 Tables)** using HR-LCMS, GC-MS, and HPLC methods. Their 3D structures were retrieved from the PubChem database in SDF format (https://pubchem.ncbi.nlm.nih.gov/) (Accessed on 14 July 2022). The retrieved compounds were further screened for their pharmacokinetics parameters which include ADME property using multiple parameters such as oral bioavailability (OB) ≥ 30%, druglikeness (DL) ≥ 0.18, Lipinski’s rule of five, clearance (CL) > 15 mL, half-life (HL) < 3 h, molecular weight (MW), hydrogen bond donors (HbD), hydrogen bond acceptors (HbA), topological polar surface area (TPSA). The compounds were selected chiefly on the basis of their reliability for oral consumption. It was also taken into consideration that the compounds should not allergenic or mutagenic. The compounds which pass all the above parameters were selected for network construction [21].

### Identification of target and disease-associated genes

The core target genes of the compounds which pass the pharmacokinetics criteria were retrieved from Swiss Target Prediction (http://www.swisstargetprediction.ch/) (Accessed on 14 July 2022). Using a compound that is presumed to be bioactive, this web tool enables users to calculate the most likely macromolecular targets. Furthermore, to build the compound-target-disease network, the identification of genes associated with hyperglycemia and obesity was important. The genes related to both diseases were retrieved using DisGeNET database (https://www.disgenet.org/search) (Accessed on 14 July 2022) and GeneCards database (https://www.genecards.org/) (Accessed on 14 July 2022). The DisGenNet is a free-to-access platform that is intended to address a number of issues regarding the genetic causes of human diseases, whereas GeneCards is a database of human genes that offers genomic, proteomic, transcriptomic, genetic, and functional details on every human gene known or projected. The platform is made to answer a variety of questions about the genetic causes of human disorders. A thorough search was conducted using both the platforms using the keywords “hyperglycemia” and “obesity”, using “*Homo sapiens*” as a filter. A combination of results obtained from both platforms was used for further analysis [21].

### PPI network construction

Based on the core active genes of the compound targets and disease-related genes were uploaded to the STRING database (https://string-db.org/) (Accessed on 14 July 2022) to construct the PPI network. STRING is a biological database and online resource for known and anticipated protein-protein interactions in molecular biology. Information is available in the STRING database from a variety of sources, including experimental data, computer prediction techniques, and publicly available text collections. The network was generated with "*Homo sapiens*" in consideration, and the confidence level was set to the greatest possible value (>0.9). Proteins are represented by the network’s nodes, while related proteins are represented by their edges [21].

### C-T (compound-target) network and enrichment analysis

The obtained protein-protein network to Cytoscape 3.8.2. It is an open-source bioinformatics software platform for visualizing molecular interaction networks and integrating with gene expression profiles and other state data. Plugins with additional functions are available. By using the CytoCluster plugin, the gene cluster was obtained to find the highly significant genes. Later, the core targets were selected using the most important parameters based on the topological characteristics of the constructed networks, such as Degree Centrality (DC), which is used to evaluate the number of nodes associated with a node, Closeness Centrality (CC), which refers to the sum from one point to another, and Betweenness Centrality (BC), which is used to evaluate the shortest path in the network using the CytoNCA plugin. Finally, the selected genes were again screened using the CytoHubba plugin using the Maximum Clique Centrality (MCC) scoring method to generate the critical subnetwork. The biological interpretation of core target genes was performed using GO and KEGG pathways analysis to creates a functionally organized network, further using DAVID to identify the potential biomarkers that are associated with the pathway, as well as the cellular location of the core genes, analyzed. Finally, the C-T-D network was built for the core targets and was analyzed using Cytoscape 3.8.2 [21].

### Molecular docking simulation

The crystal structures of protein molecules for MAPK3 (PDB ID: 2zoq) were retrieved from the RCSB PDB database (https://www.rcsb.org/) (Accessed on 16 July 2022). The preparation of protein molecules was done according to the previous study by the authors using AutoDock Tools 1.5.6 [22, 23]. To purify the protein structure, water and heteroatoms were removed. On the other hand, polar hydrogens were added to stabilize the same. The energy of the protein structure was reduced by using Kollmann-united charges and Gasteiger charges. After energy minimization, all atoms were assigned an AutoDock 4 atom type before obtaining the prepared protein structure in PDBQT format for molecular docking simulation. Further, the binding site prediction was done using the literature available [24]. The grid box measuring 40 Å × 40 Å × 40 Å containing the binding pocket was positioned at x = 29.005 Å, y = 7.191 Å, and z = 18.249 Å was created. During ligand preparation, phytochemical structures were prepared for the molecular docking simulation using AutoDock Tools 1.5.6., according to a previous study by the authors, where the 3D SDF structures were converted to PDBQT format, and applied with the Kollmann-united charges and Gasteiger charges. After energy minimization, ligand molecules were saved in PDBQT format in the same directory as the protein molecule to carry out the docking simulation [25, 26].

The virtual screening of the compounds was completed with a command-line-based software known as AutoDock Vina 1.1.2. It uses the Broyden-Fletcher-Goldfarb-Shanno (BGFS) algorithm to perturb and allocate ligands into the target site, and analyses the scoring function of each ligand conformation [27]. Because of the large number of torsions produced during ligand formation, ligands were considered to be flexible throughout the docking simulation, whereas protein was assumed to be rigid. For ligand molecules, however, 10 degrees of freedom were allowed. Out of ten binding poses generated, the first one with zero root-mean-square deviation (RMSD) of atomic positions is considered to be extremely genuine. It also has the strongest binding affinity of any position, indicating that the binding is more effective. The visualization of molecular docking simulation was completed using Biovia Discovery Studio Visualizer 2021, an open-source visualizing GUI software. The extent of ligand interaction was determined using binding affinity, the total number of intermolecular bonds, and the total number of hydrogen bonds [28].

### Molecular dynamics simulations

Docked complexes of protein with chosen ligands along with metformin as standard were selected for the molecular dynamics (MD) simulation. The MD simulation was run using the biomolecular software package of GROMACS-2018.1, according to the previous study conducted by the authors [29]. GROMACS is a comprehensive software package for performing molecular dynamics or simulating Newtonian equations of motion for systems containing hundreds to millions of particles. It is primarily intended for biological compounds with several complex bound connections, such as proteins, lipids, and nucleic acids. The software is exceptional at computing nonbonded interactions, which are frequently seen as the most important in simulations. The CHARMM36 force field (https://www.charmm.org/archive/charmm/resources/charmm-force-fields/) (Accessed on 17 July 2022) was used to approximate the ligand structures, and the SwissParam server (https://www.swissparam.ch/) (Accessed on 17 July 2022) was used to construct the ligand topology [30–32]. On the other hand, using the pdb2gmx module, protein structure was also added with the CHARMM36 forcefield. The next step involved 5000 steps of energy minimization in the vacuum using the steepest descent approach. The distance between each protein complex and the box’s edges was 10 Å. To maintain the necessary 0.15 M salt concentration, the solvent was incorporated into the TIP3P water model with the proper number of Na^+^ and Cl^-^ counterions. In total, 5 simulations were run for 100 ns simulation time at 310K temperature and 1 bar pressure. The trajectory analysis of root-mean-square deviation (RMSD), root-mean-square-fluctuation (RMSF), the radius of gyration (Rg), solvent accessible-surface-area (SASA), and ligand hydrogen bond parameters was done and the results were plotted in the graphical format using XMGRACE software, a GUI based software used for plotting the results of MD simulation [33].

### Binding free energy calculations

Outcomes of the MD simulation run for complexes along with standard were subjected to binding free energy calculations using the Molecular Mechanics/Poisson-Boltzmann Surface Area (MM-PBSA) technique. It is another application of molecular dynamics simulations and thermodynamics for determining the extent of ligand binding with protein. The g_mmpbsa program with MmPbSaStat.py script, which utilizes the GROMACS 2018.1 trajectories as input, was used to determine the binding free energy for each ligand-protein combination [34, 35]. In the g_mmpbsa program, three components are used to calculate the binding free energy: molecular mechanical energy, polar and apolar solvation energies, and molecular mechanical energy. The calculation is done using MD trajectories of the last 50 ns were considered to compute ΔG with dt 1000 frames. It is evaluated using molecular mechanical energy, and polar-apolar solvation energies. The Eqs (I) and (II) that are used to calculate the free binding energy are given below.


$$\Delta G_{Binding}=G_{Complex}-(G_{Protein}+G_{Ligand})$$ (I)

$$\Delta G=\Delta E_{MM}+\Delta G_{Solvation}-T\Delta S=\Delta E_{\left(Bonded+non‐bonded\right)}+\Delta G_{\left(Polar+non‐polar\right)}-T\Delta S$$ (II)

G_Binding_: binding free energy, G_Complex_: total free energy of the protein-ligand complex, G_Protein_ and G_Ligand_: total free energies of the isolated protein and ligand in solvent, respectively, ΔG: standard free energy, ΔE_MM_: average molecular mechanics potential energy in vacuum, G_Solvation_: solvation energy, ΔE: total energy of bonded as well as non-bonded interactions, ΔS: change in entropy of the system upon ligand Binding, T: Temperature in Kelvin [36, 37].

### PASS activity prediction and ADMET profiling

From the previous analysis, details regarding the pharmacokinetics and druglikeness analysis of the representative compounds (caffeic acid, DBDEA, syringic acid, and metformin) were obtained (virtual screening using molecular docking and ADMET profiling). Additionally, the representative compounds were subjected to pharmacological activity prediction using the PASS online program. The PASS server assesses whether the chemical compound(s) offered can have a certain pharmacological effect. The results were numerical and divided into "Pa" and "Pi," where "Pa" stands for potential activity and "Pi" for potential inactivity of the given molecule. The compounds that are deemed appropriate for a certain pharmacological activity have relative Pa values that are higher than Pi values (Pa > Pi). In this study, the "MAPK inhibitor activity" parameter was selected for the PASS analysis [38].

## Results and discussion

### Screening of bioactive compounds from library and target prediction

**Fig 1:**
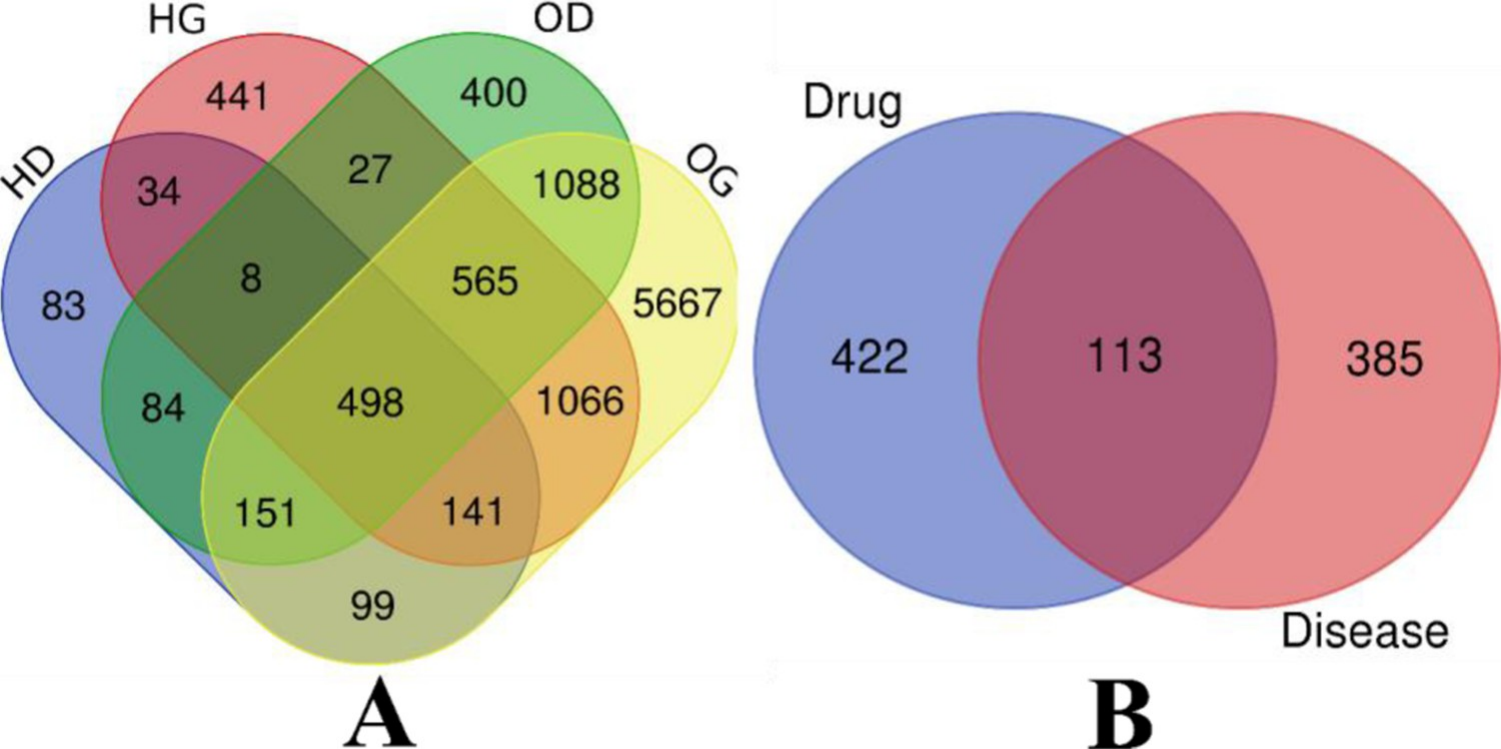
Venn diagram representing the (A) genes related to hyperglycemia with obesity by the union of DisGeNet and GeneCards. (B) identification of drug-target disease-related genes. *Note*: H: Hyperglycemia, O: Obesity, D: DisGeNet, G: GeneCards.

A total of 108 active compounds were screened on the bases of ADME properties, among which 13 core active compounds were selected based on the screening criteria as shown in **Table 1.** Compounds with high bioavailability will be more efficient since they enable the body to absorb more of the necessary nutrient without requiring greater doses. Druglikeness (DL) gauges how likely it is for a molecule to be bioavailable as an oral drug. In the early stages of drug discovery, DL generated from the structures and characteristics of currently available medications and drug prospects has been routinely employed to screen out unwanted molecules [39, 40]. Since our aim was to find orally consumable bioactive compounds with no adverse effects like mutagenicity and allergenicity, the criteria used in our previous work for the ADME-based screening were used [21]. Further, based on the core active compounds their key targets were identified from the SwissTargetPrediction database (http://www.swisstargetprediction.ch/) (Accessed on 14 July 2022). A total of 1076 target genes of each active compound were retrieved. Additionally, genes related to disease were retrieved from DisGeNET and GeneCards databases, a total of 498 genes were considered as they are common in hyperglycemia and obesity **(Fig 1).**

**Table 1:** Screening of active compounds using ADMET criteria.

Sl. No	Compound Names	PubChem CID	Oral bioavailability (OB≥30%)	Druglikeness (DL≥ 0.18)	Lipinski’s rule of five (LR)	Clearness (CL 5 ≥ 15 ml/min/kg)	Drug half-life (HL<3h)	Molecular Weight (MW 100–600)	Hydrogen bond acceptor (0~12)	Hydrogen bond donor (0~7)	TPSA (0–140)
1	Dihydroquercetin	439533	Moderate	2.30	Accepted	9.517	0.850	304.060	7	5	127.450
2	Tetrahydrosappanone A trimethyl ether	6708784	Pass	1.35	Accepted	11.315	0.478	330.150	5	1	57.150
3	7-Hydroxyetodolac	14112821	Pass	1.68	Accepted	6.427	0.830	303.150	5	3	82.550
4	Deoxysappanone B 7,3’-dimethyl ether acetate	6708755	Pass	1.32	Accepted	6.435	0.318	356.130	6	0	71.060
5	Cortol	246873	Moderate	0.49	Accepted	11.072	0.202	368.260	5	5	101.150
6	Tiamulin	656958	Pass	1.31	Accepted	15.755	0.358	493.320	5	1	66.840
7	p-γ-Dihydroxyphenylbutazone	4781	Pass	6.74	Accepted	12.780	0.882	340.140	6	3	89.870
8	Didesmethylimipramine	159642	Moderate	0.45	Accepted	10.188	0.085	252.160	2	2	29.260
9	Trimipramine	5584	Pass	5.25	Accepted	10.654	0.045	294.210	2	0	6.480
10	(1-Cyclopropylethyl) methylamine	16772299	Pass	2.81	Accepted	10.507	0.495	99.100	1	1	12.030
11	Stigmasta-4,6,22-trien-3beta-ol	91744920	Moderate	1.03	Accepted	15.413	0.026	410.350	1	1	20.230
12	Caffeic acid	689043	Moderate	1.62	Accepted	10.973	0.930	180.040	4	3	77.760
13	Syringic acid	10742	Pass	1.99	Accepted	7.208	0.946	198.050	5	2	75.990

### C-T-D network construction

**Fig 2:**
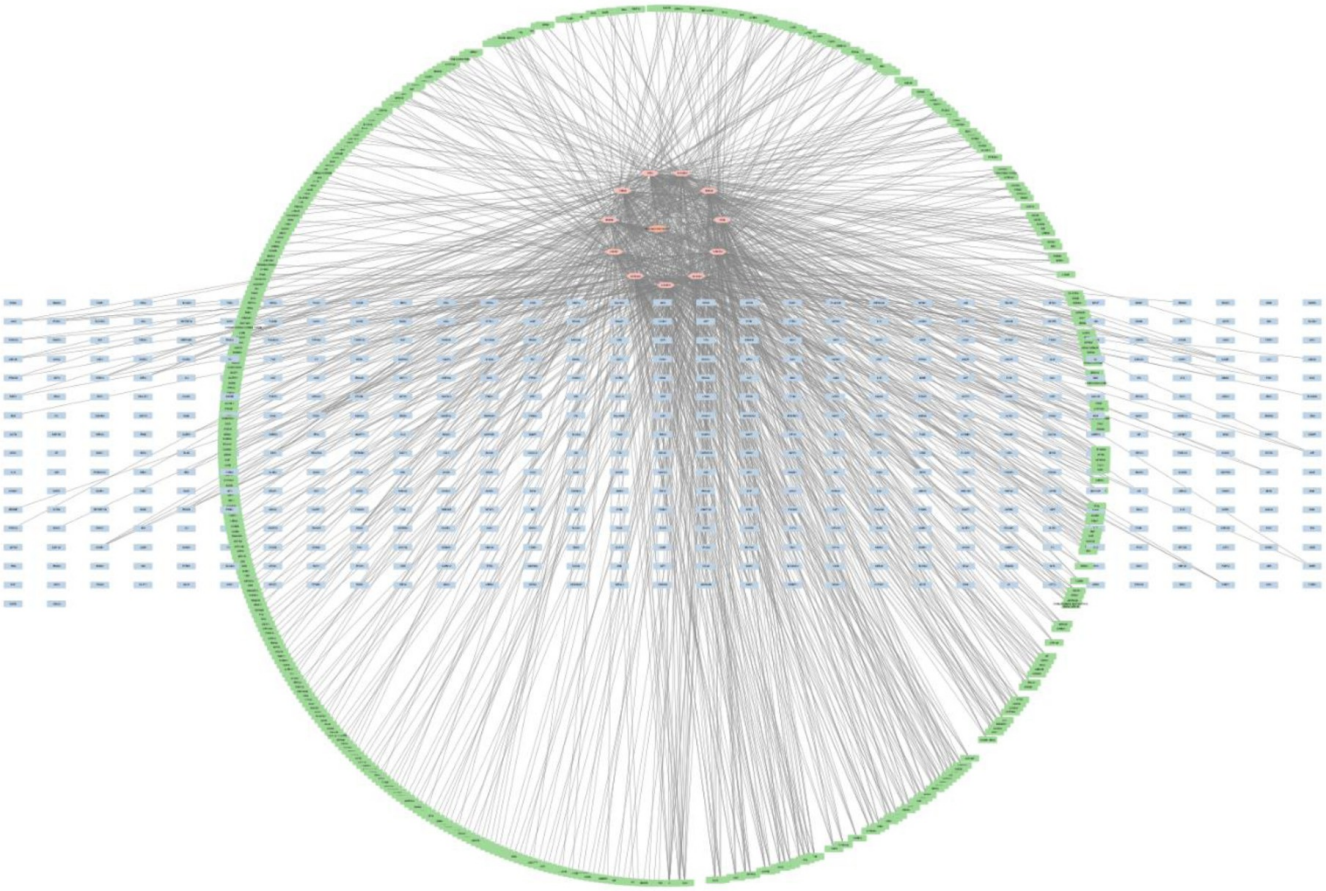
Construction C-T-D network. The pink colour represents compounds. The green colour represents targets related to the compounds and blue represents disease-related genes.

**Fig 3:**
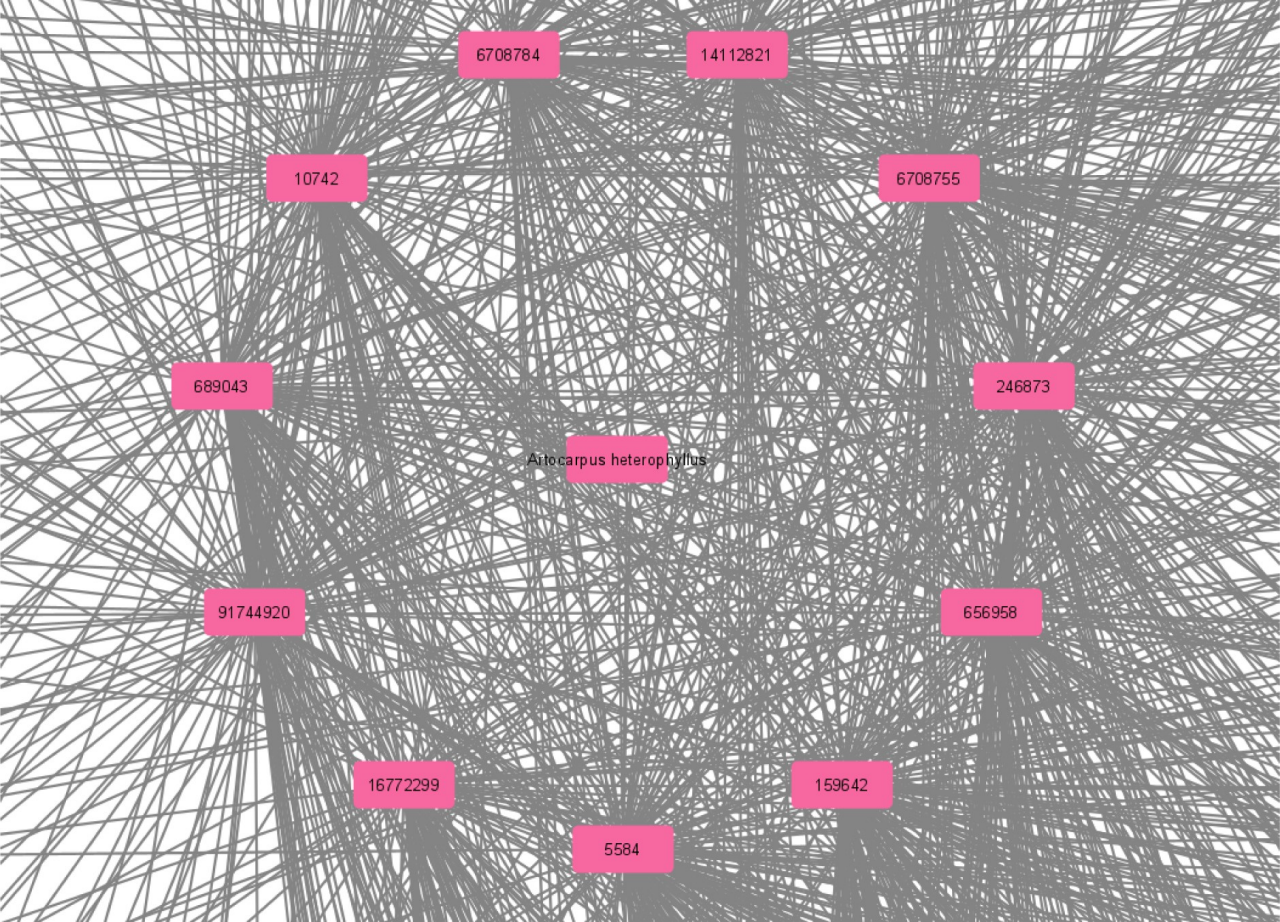
Identified 11 hit compound ID’s linking with the targets of obesity-linked DM. The names of the corresponding phytochemicals have been given in Table 1.

**Fig 4:**
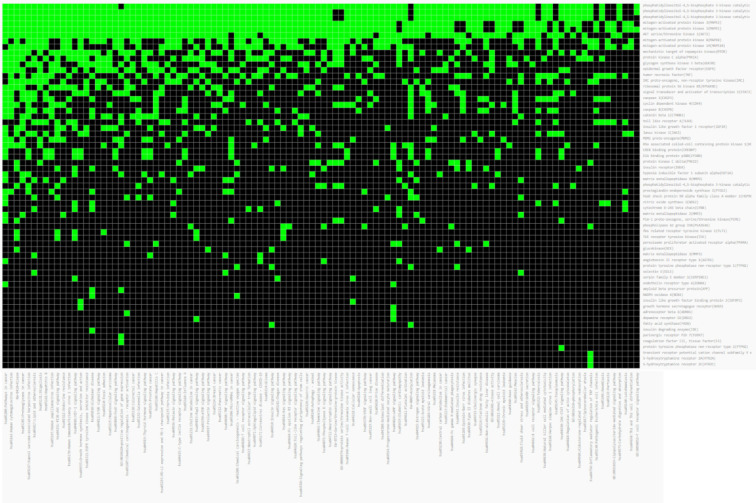
GO and KEGG enrichment analysis.

A total of 498 disease-related genes were mapped to 1076 target-related genes as shown in **Fig 2** to get a better understanding of the C-T-D. According to the analysis, a total of 932 nodes and 1087 edges were present. Based on the network it can be seen that each compound is connected to more than one target, which may indicate that the compound may participate in the treatment of obesity-linked DM. The compounds corresponded to multiple targets. This is a strong indication that many targets may induce a synergistic effect when *A*. *heterophyllus* serves as an anti-diabetic as well as an anti-hyperlipidemic agent. Out of the 13 compounds selected from ADME-based screening, 11 were found to be linked with the targets **(Fig 3)**. Except for dihydroquercetin and p-γ-dihydroxyphenylbutazone, all the other 11 compounds were found to have a link with the targets. The name and PubChem CIDs of the selected compounds have been given in **Table 1**. The targets were associated with protein phosphorylation, insulin receptor, insulin-like growth factors and so on, according to GO functional analysis. The KEGG pathway analysis was used to determine the key signaling pathways associated with the anti-T2DM action. The majority of the genes were found to be involved in the following pathways: cancer pathways, neuroactive ligand-receptor interactions, insulin pathways, and infectious pathways **(Fig 4)**.

### PPI network analysis and enrichment analysis

**Fig 5:**
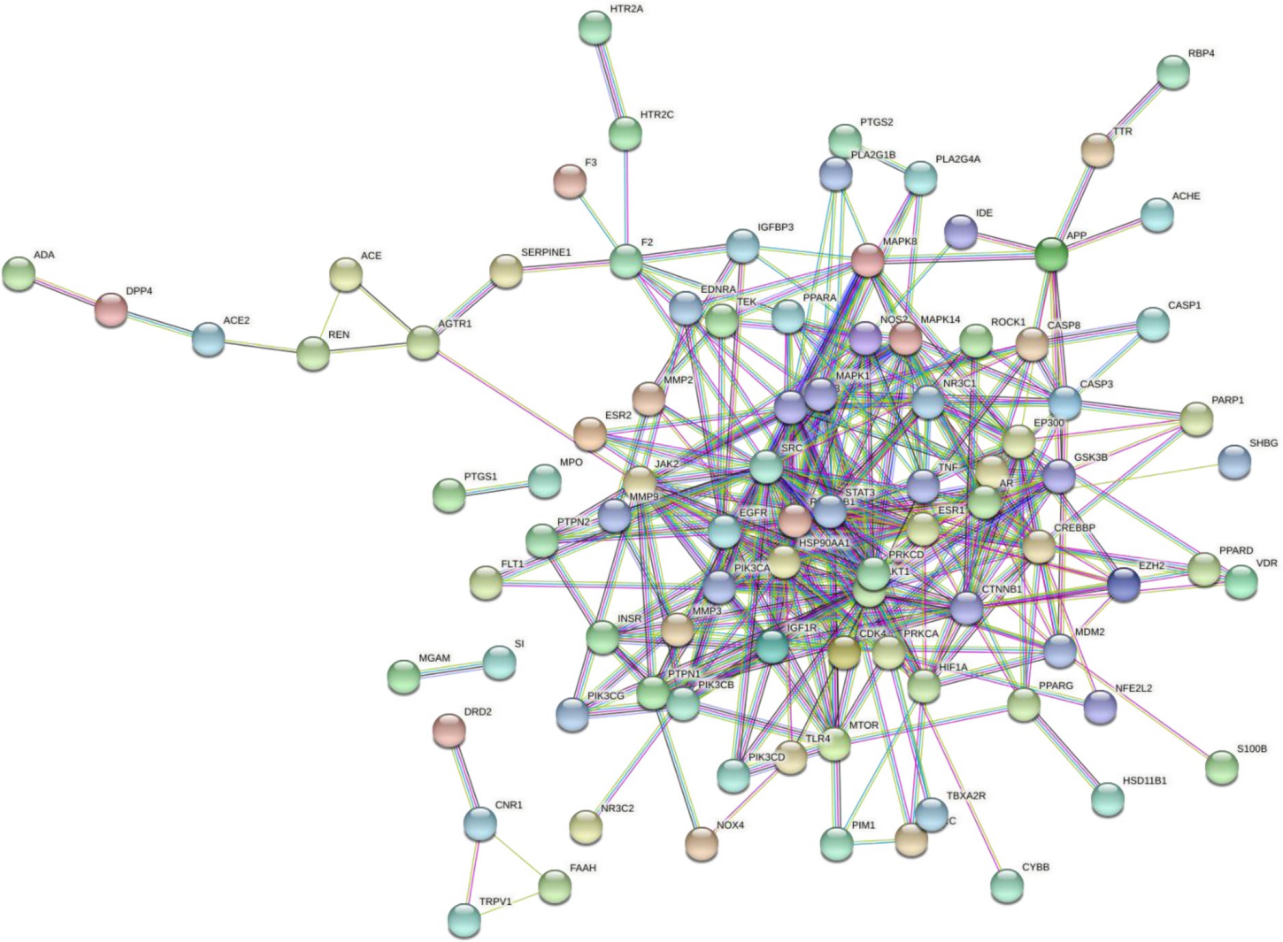
PPI network of target and disease-related genes constructed using STRING database.

**Fig 6:**
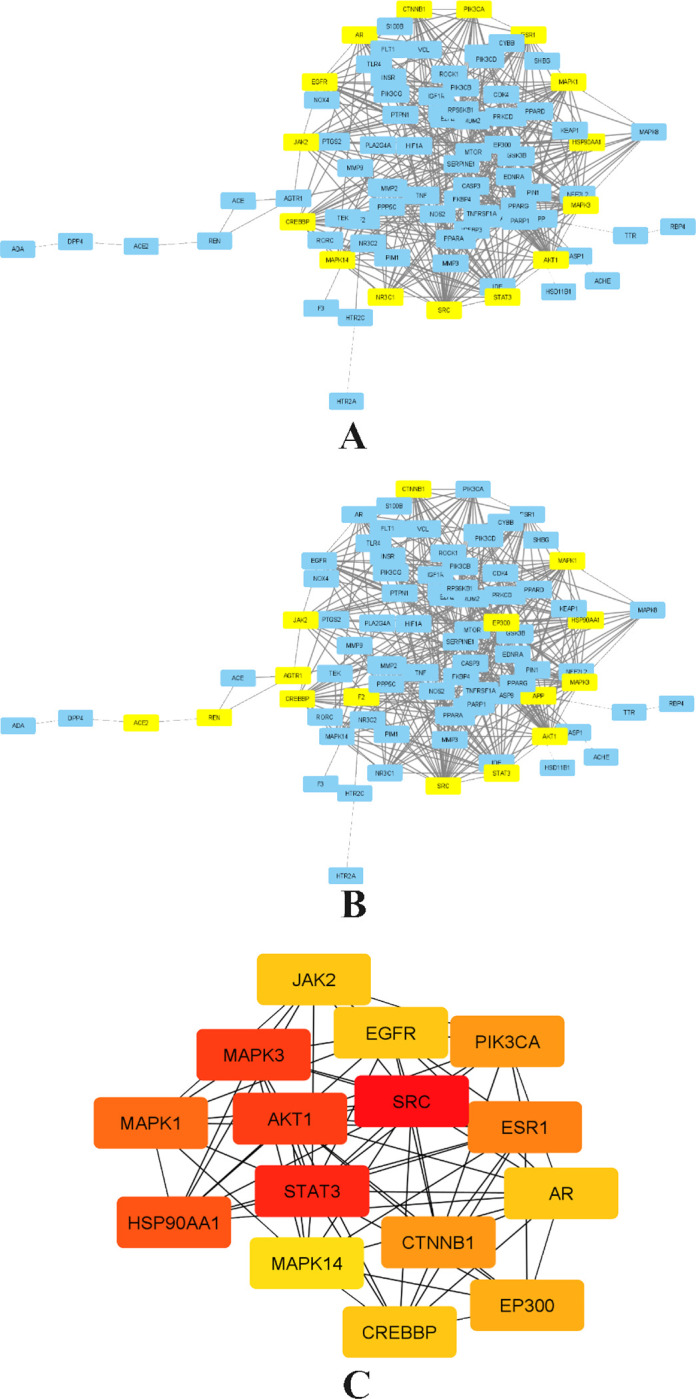
The topological screening for identifying subnetwork network. The PPI network diagram of 15 core targets was obtained by screening 113 targets. A) First filtration of network by CytoNca, the yellow nodes were screened with each score higher than median. B) second subnetwork filtration via CytoNca. The yellow nodes were screened with a score higher than the median. C) core key subnetwork of top 15 nodes analyzed by CytoHubba.

The interaction network was constructed for the overlapping genes between the diseases and the drug targets, the network was constructed for 113 overlapping targets that were specific to Homo sapiens. The constructed network contained 113 nodes and 332 edges. The high confidence target proteins i.e., greater than the score of 0.9 were selected, and the unconnected proteins from the network were removed. The highest degree indicates that the targeted genes have a strong correlation with one another; as a result, all of these genes may be important targets [21]. These results are then contrasted with the results of enrichment analysis. The diagrammatical representation of the PPI network is given in **Fig 5**. The network was later imported to Cytoscape 3.8.2. to investigate the critical subnetwork. The cluster analysis of the network was performed based on betweenness, closeness, and degree according to the score calculated by CytoNca, and CutyoHubba **(Fig 6)** the top 15 targets were screened based on the value of genes that were larger than the median in all results. According to the network analysis, the insulin signaling pathway has been predicted to have the strongest affinity to MAPK3 protein, which was chosen as the target [24].

### Molecular docking simulations

The selected phytocompounds (11) from whole green jackfruit flour methanol extract (JME) were virtually screened against the target enzyme human MAPK3 (PDB ID: 2ZOQ), and the results revealed that every single molecule was bound to the inhibitor binding site of the enzyme. They were found to bind with the c-helix region, which is known for its catalytic activity (phosphorylation). Binding in this region would inhibit the key residues from getting activated. Therefore, binding of the phytocompounds in this region could reduce the enzyme activity [24]. While there were several intermolecular interactions, including hydrogen bonds, compounds including caffeic acid, deoxysappanone B 7,3’-dimethyl ether acetate (DBDEA), and syringic acid were predicted to have the highest binding affinity (better estimated free energy of binding). The above-mentioned compounds were chosen for additional in silico analysis because they meet the aforementioned criteria for pharmacological target, in contrast to metformin and other phytocompounds from JME. Results from the virtual screening of selected phytocompounds of JME against the target enzyme human MAPK3 have been given in **Table 2**.

**Table 2:** Virtual screening results selected phytocompounds of JME docked with the human MAPK3.

Sl. No	Compound Names	BA	TIN	HB
1	(1-Cyclopropylethyl)methylamine	-4.1	4	1
2	7-Hydroxyetodolac	-5.9	4	2
**3**	**Caffeic acid**	**-8.0**	**7**	**3**
4	Cortol	-7.4	5	-
**5**	**Deoxysappanone B 7,3’-dimethyl ether acetate**	**-8.2**	**11**	**3**
6	Didesmethylimipramine	-6.0	4	1
7	Stigmasta-4,6,22-trien-3beta-ol	-8.1	3	-
**8**	**Syringic acid**	**-8.5**	**12**	**5**
9	Tetrahydrosappanone A trimethyl ether	-7.5	4	1
10	Tiamulin	-5.6	2	1
11	Trimipramine	-7.6	10	-
12	Metformin	-5.2	2	2

*Note*: BA: binding affinity in kcal/mol, TIN: total number of intermolecular interactions, THB: total number of hydrogen bonds.

**Fig 7:**
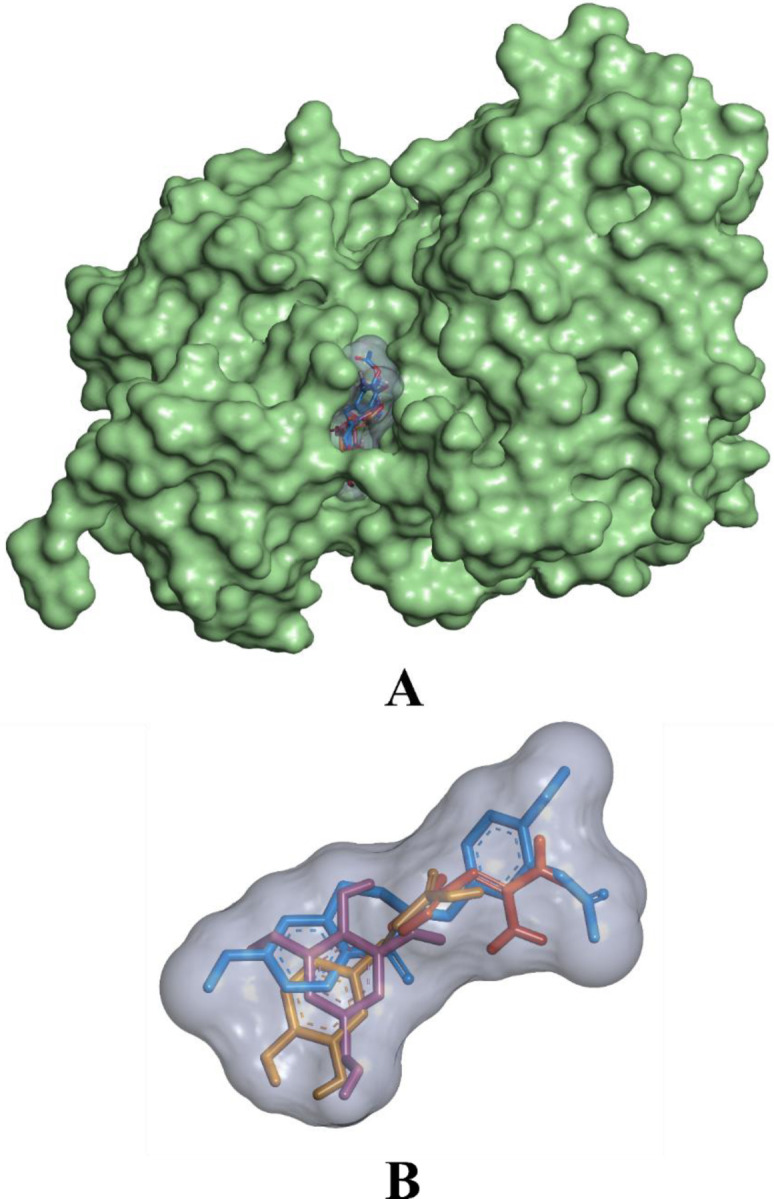
Binding of phytocompounds from JME and metformin inside the inhibitor binding site of human MAPK3 in 3D. A) surface representation of phytocompounds from JME binding into the active site, B) phytocompounds from JME and metformin in the binding pocket. Orange: caffeic acid, blue: DBDEA, purple: syringic acid, red: metformin.

**Fig 8:**
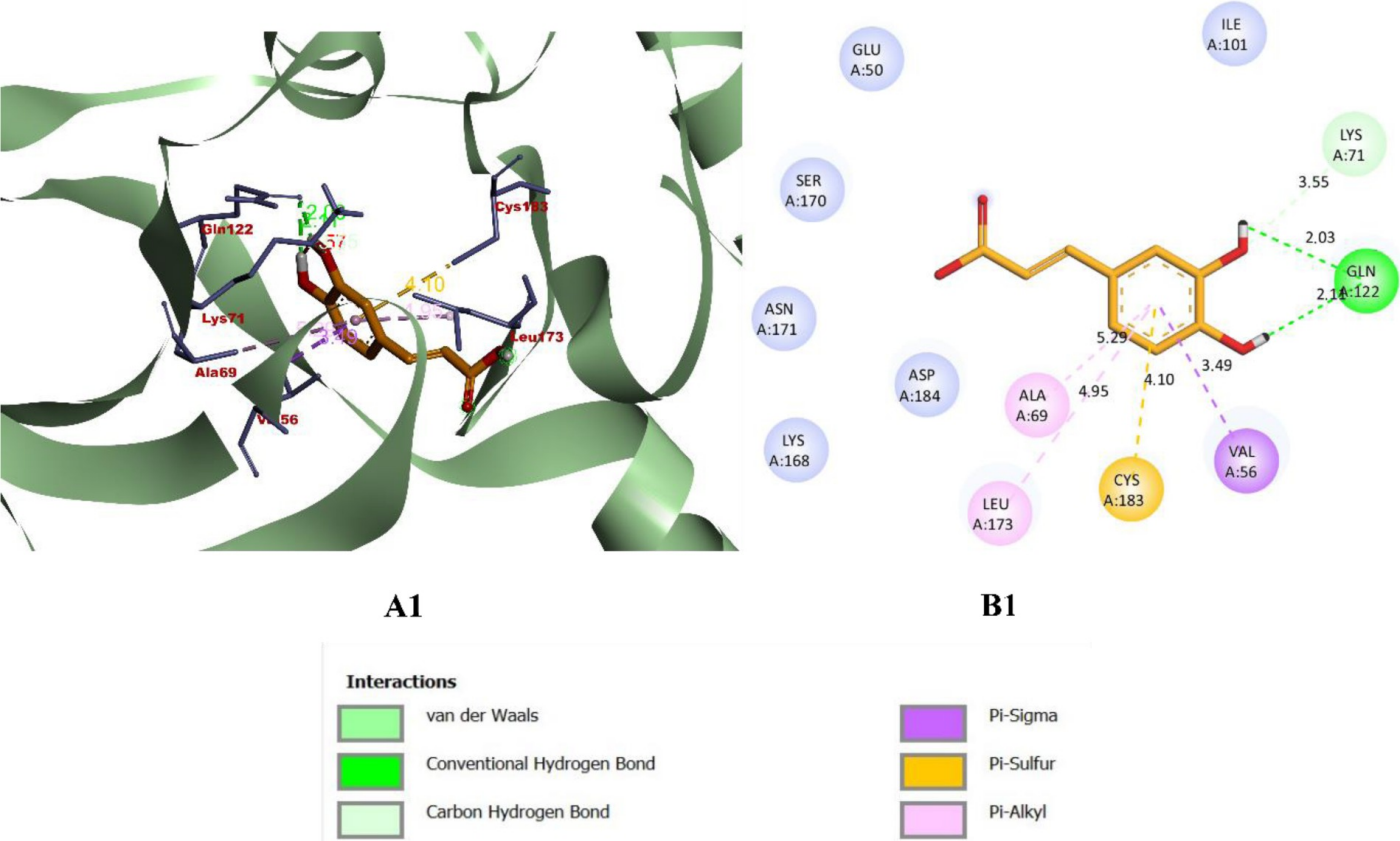
Arrangement (A1) and binding pattern (B1) of binding interactions of caffeic acid from JME with the residues of human MAPK3 protein.

**Fig 9:**
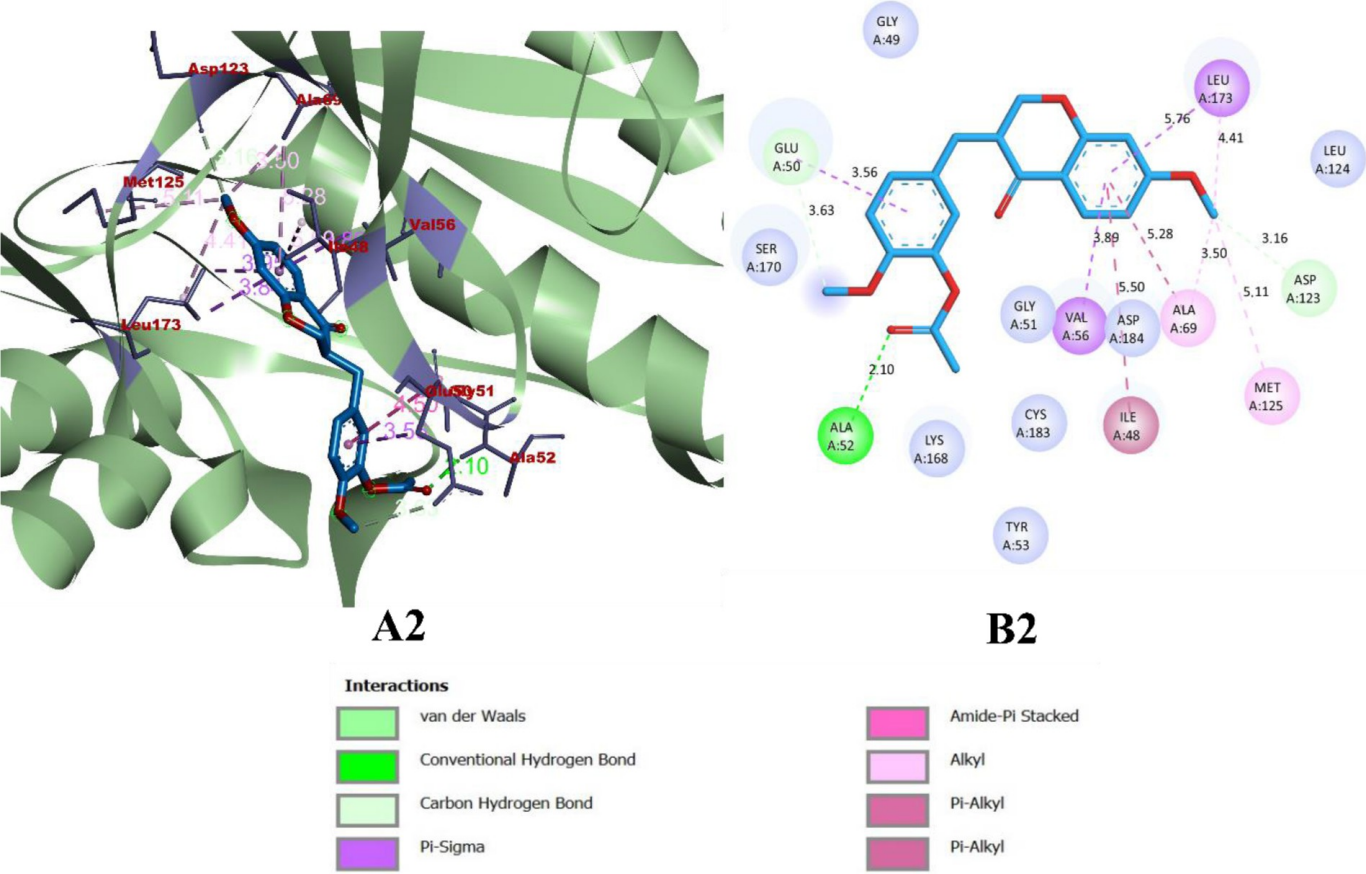
Arrangement (A2) and binding pattern (B2) of binding interactions of DBDEA from JME with the residues of human MAPK3 protein.

**Fig 10:**
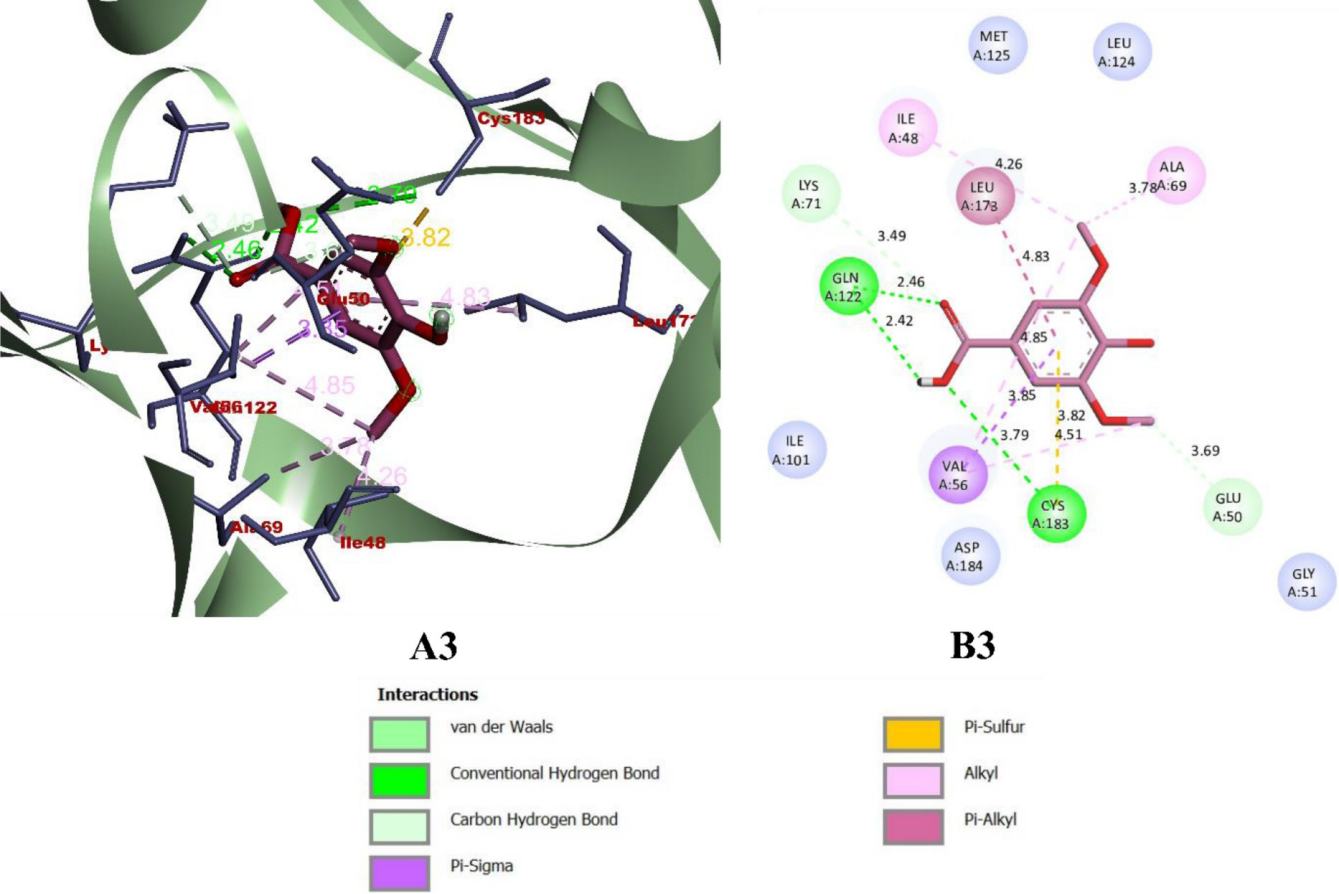
Arrangement (A3) and binding pattern (B3) of binding interactions of syringic acid from JME with the residues of human MAPK3 protein.

**Fig 11:**
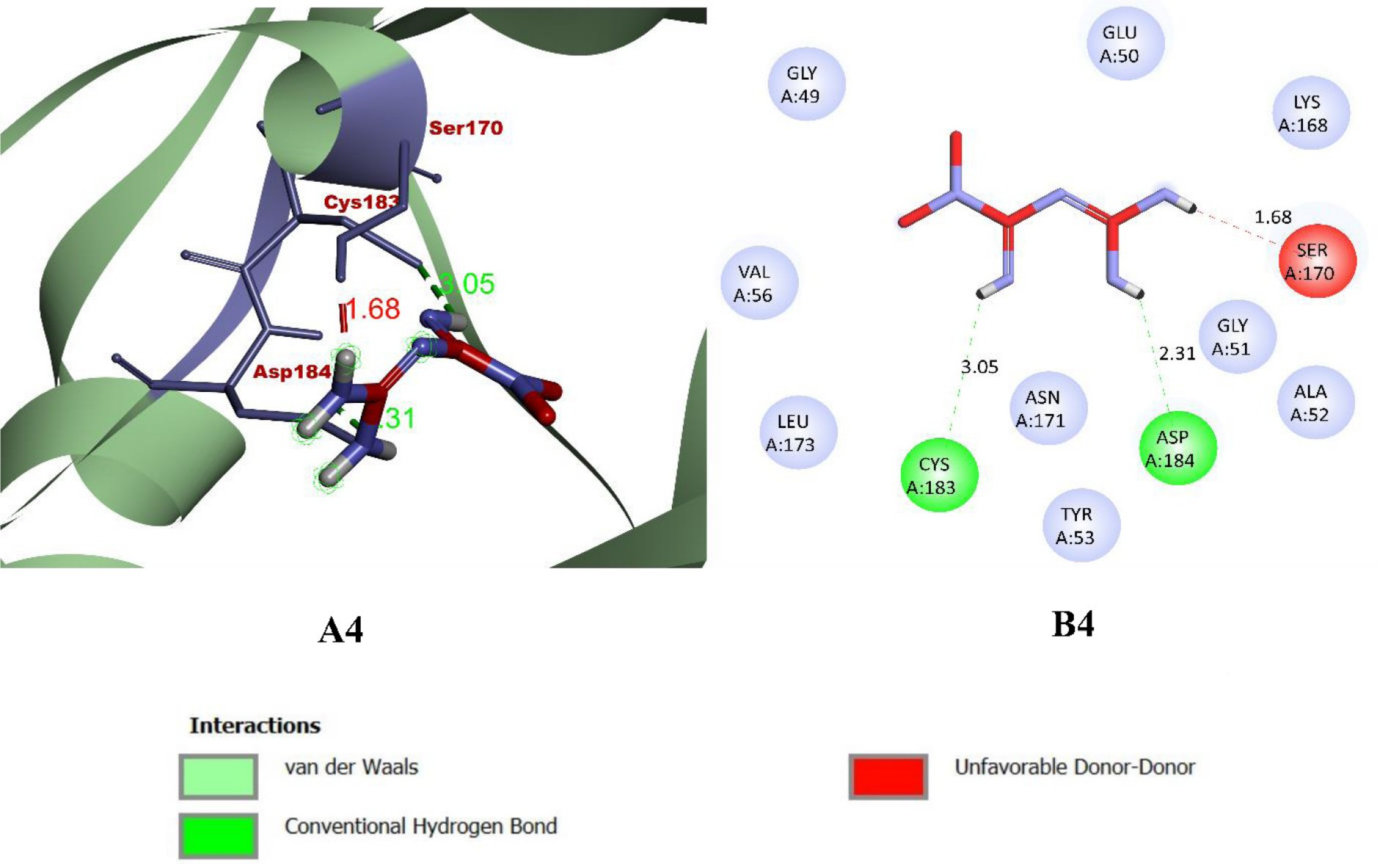
Arrangement (A4) and binding pattern (B4) of binding interactions of metformin from JME with the residues of human MAPK3 protein.

**Fig 12:**
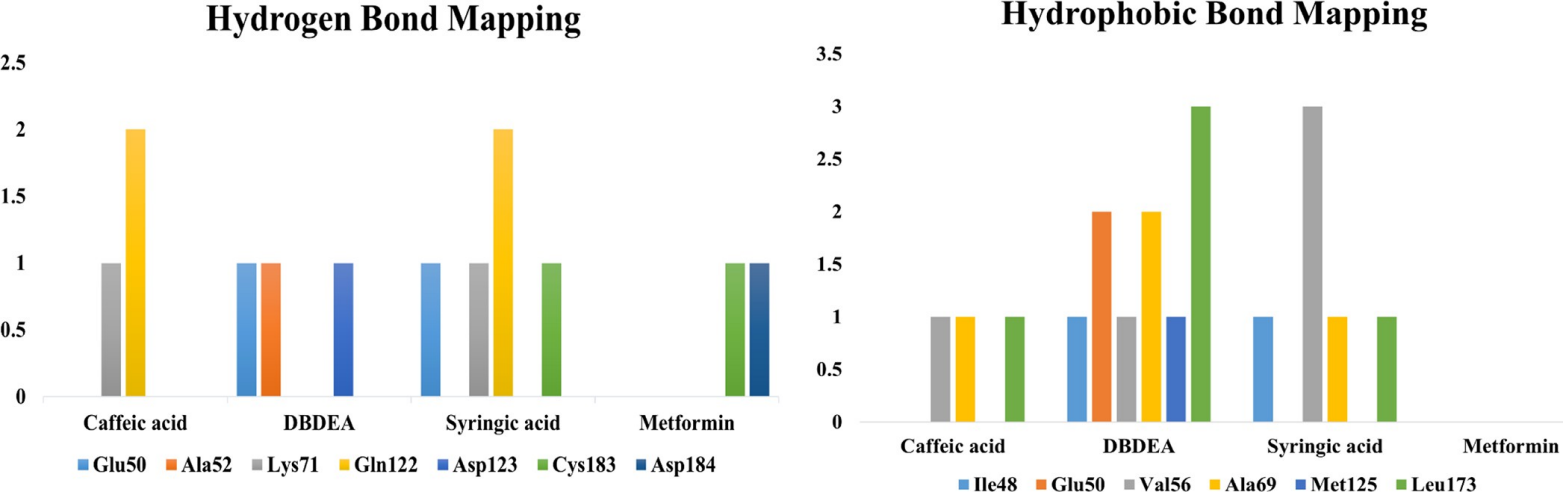
Hydrogen and hydrophobic bond mapping of the experimental compounds docked with human MAPK3 protein.

While binding with the inhibitor binding site (c-helix) of human MAPK3, caffeic acid, DBDEA, syringic acid, and metformin were found to be docked deep inside the binding pocket, occupying the cleft present in the inhibitor binding site **(Fig 7)**. The compound caffeic acid was predicted to form a total of 7 intermolecular interactions, with 3 of them being hydrogen bonds. With these interactions, the binding affinity of caffeic acid was found to be -8.0 kcal/mol. Compound DBDEA had 11 intermolecular bonds and 3 hydrogen bonds with a binding affinity of -8.2 kcal/mol. Among the compounds docked, syringic acid has the highest binding affinity (-8.5 kcal/mol) with 12 intermolecular bonds and 5 hydrogen bonds. However, metformin was not predicted with better binding efficiency. The control drug formed a total of 2 intermolecular interactions, both of them being hydrogen bonds. It also formed a donor-donor unfavorable hydrogen bond with Ser170 (1.70 Å). The donor-donor hydrogen bond indicates the unlikeliness of the ligand and protein to form a bond since both entities try to donate the atom for bonding. Though not significantly, this type of bond negatively affects the binding affinity, since they are unlikely to form. With these binding interactions, the compound was predicted with a binding affinity of -5.2 kcal/mol. The results of the docking simulation indicate that molecules from JME can bind inside the binding site of the target enzyme and can induce biological activity. **Figs 8–11** visualize the arrangement and binding pattern of binding interactions of selected phytocompounds from JME (caffeic acid, DBDEA, and syringic acid, respectively) and metformin with the residues of human MAPK3 protein, whereas **Table 3** gives an account of them along with the bond length. During the binding interaction, Gln122 was found to be the most favorable residue for the hydrogen bond, since caffeic and syringic acid formed 2 hydrogen bonds each with it **(Fig 12)**. In case of hydrophobic bond mapping, Leu173 and Val56 were found to be the most favorable and were chiefly bound to DBDEA and syringic acid. Therefore, it could be concluded that Gln122, Leu173, and Val56 of the inhibitor binding site could act as key residues to inhibit the protein activity.

**Table 3:** Binding interactions of compound 6b and acarbose with α-glucosidase along with their bond length (Å).

Compounds	Binding affinity (kcal/mol)	Hydrogen bonds	Hydrophobic bonds				Other bonds
			Pi-pi	Pi-alkyl	Pi-sigma	Alkyl	Pi-sulfur
Caffeic acid	-8.0	Gln122 (2.03), Gln122 (2.11), Lys71 (3.55)	-	Ala69 (5.28), Leu173 (4.94)	Val56 (3.48)	-	-
DBDEA	-8.1	Ala52 (2.10), Glu50 (3.62), Asp123 (3.15)	Glu50 (4.49)	Ile48 (5.44), Ala69 (5.28)	Glu50 (3.56), Val56 (3.89), Leu173 (3.94), Leu173 (3.83)	Ala69 (3.50), Met125 (5.11), Leu173 (4.41)	-
Syringic acid	-8.5	Gln122 (2.46), Cys183 (3.78), Gln122 (2.42), Lys71 (3.48), Glu50 (3.69)	-	Leu173 (4.83)	Val56 (3.84),	Ala69 (3.77), Ile48 (4.25), Val56 (4.85), Val56 (4.51)	Cys183 (3.82)
Metformin	-5.2	Asp184 (2.30), Cys183 (3.04)	-	-	-	-	-

The binding interactions were similar and the docking was accurate, according to a previous study by **Noor et al. (2022)**, where selected phytochemicals from *Abrus precatorius* were docked into the human MAPK3 inhibitor binding site (PDB ID: 2ZOQ) [41]. Since the phytochemicals like abrisapogenol J, precatorine, sophoradiol, and others were found to interact with the key residues of the protein, they were predicted to have biological activity. Since our results are in accordance with **Noor et al. (2022)** [41], phytocompounds from our jackfruit flour could induce biological activity by interacting with the same protein molecule. In case of the chemical compounds, a few compounds from the Managed Chemical Compounds Collection (MCCC) of the University of Nottingham (UK) were found to inhibit the human MAPK3 protein, both *in vitro* and *in silico* [42]. Therefore, concerning the outcomes of both these reported studies, compounds from JME could be used for MAPK3 inhibition.

### Molecular dynamics simulations

In addition to the docking, molecular dynamic (MD) simulation studies were performed to explain the dynamic behavior of the protein-ligand complex with respect to time under a solvated environment. The simulation study provides the analysis of protein-ligand complex RMSD, the Rg, SASA, ligand RMSD, the total number of ligand hydrogen bonds maintained throughout the simulation time, and the variation of secondary structure pattern between the protein and their complexes [37, 38]. The RMSD of the protein-ligand complex depicts the stability of the same throughout the simulation by determining the presence of a ligand inside the binding pocket. The Rg considers the varied masses calculated to root mean square distances considering the central axis of rotation. It considers the capability, shape, and folding during each time step on the whole trajectory throughout the simulation. RMSF concentrates on the protein structural regions that differ the most/least from the mean. SASA measures the area around the hydrophobic core formed between protein-ligand complexes. Further, ligand hydrogen bonds appear during the molecular docking study being analyzed over the total simulation period. All the intermolecular hydrogen bonds between the ligands and the respective protein only were considered during the analysis and plotted accordingly [38]. In this study, 5 simulations were performed at 100 ns time with the native protein alone and in complex with the representative compounds (caffeic acid, DBDEA, syringic acid, and metformin).

In our study, all the phytocompounds from JME and metformin stayed in the inhibitor binding site till the end of the simulation run. The RMSD plots showed all the compounds including metformin gained stability after 10 ns. However, the RMSD plots of protein-caffeic complex and protein-metformin deviated from the direction of the apo-protein plot at 50 ns, indicating their instability during simulation. Therefore, protein-DBDEA and protein-syringic acid complexes have been in the binding site with more stability compared to the other 2 protein-ligand complexes. In case of RMSF, the compounds had minimal fluctuations throughout the simulation, except in the C-terminal, where higher fluctuations got detected. Protein-syringic acid complex was predicted with more fluctuations at 50–100 residues, 200, and 350 residues as well. Yet the protein-metformin complex had more fluctuations compared to the protein-DBDEA complex. Except for protein-syringic acid and protein-metformin complex, all the phytocompounds had lesser fluctuations, therefore had more stability inside the binding pocket. During the Rg analysis, all the phytocompounds were found to be compact inside the binding pocket. Since they bind inside the inhibitor binding site, the SASA got reduced for all the protein-ligand complexes. However, the SASA plots of apo-protein and protein-DBDEA complex had concurrent plots, indicating that the latter had an efficient binding capacity compared to other phytocompounds. Analysis of ligand hydrogen bonds predicted that syringic acid had a maximum of 7 hydrogen bonds, whereas DBDEA and caffeic acid had 5 and 3 hydrogen bonds, respectively.

**Fig 13:**
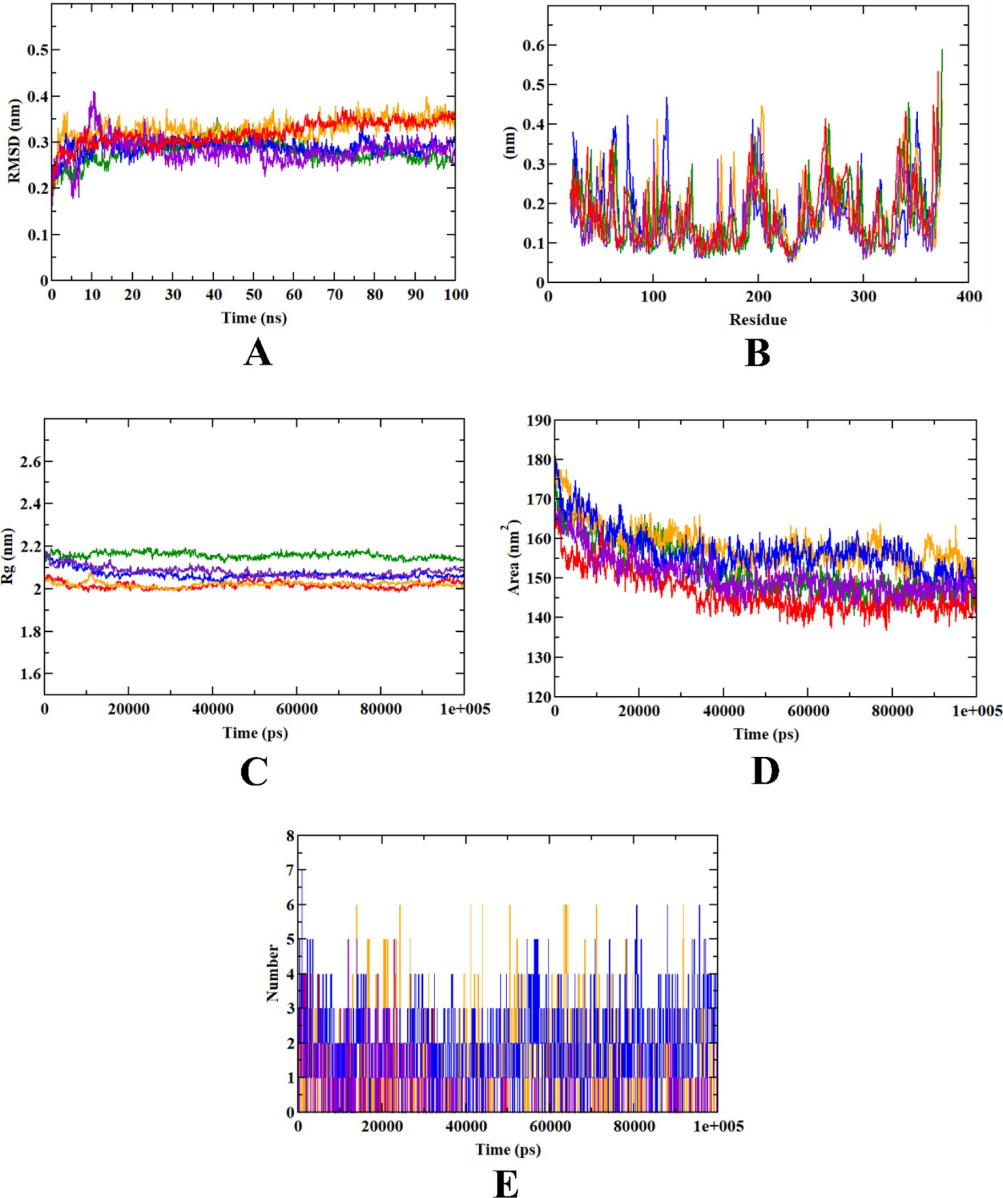
Visualization of MD trajectories of JME phytocompounds and metformin complexed with human MAPK3 protein run for 100 ns. A) protein-ligand complex RMSD, B) protein-ligand complex RMSF, C) protein-ligand complex Rg, D) protein-ligand complex SASA, E) ligand hydrogen bonds. (Green: apoprotein, orange: protein-caffeic acid complex, purple: protein-DBDEA complex, navy blue: protein-syringic acid complex, red: protein-metformin complex).

By the virtue of MD trajectory analysis, all the phytocompounds and metformin were stable inside the inhibitor binding site of the human MAPK3 protein. Although they occupy the same inhibitor binding site, values of trajectory analysis show that the DBDEA is comparatively better in comparison to other compounds. Therefore, DBDEA could be used as a potential lead inhibitor of the human MAPK3 protein. **Table 4** depicts the MD trajectory values obtained for human MAPK3 apoprotein as well as protein complexed with experimental compounds, whereas **Fig 13** visualizes the trajectory plots obtained from the simulation run.

**Table 4:** MD trajectory values of JME phytocompounds and metformin complexed with human MAPK3 protein.

MD trajectories	Apoprotein	Protein-caffeic acid complex	Protein-DBDEA complex	Protein-syringic acid complex	Protein-metformin complex
RMSD (nm)	0.25–0.30	0.35	0.25–0.30	0.30	0.35
Rg (nm)	2.1–2.2	2.0–2.1	2.0–2.1	2.0–2.1	2.0–2.1
SASA (nm^2^)	145	150	145	150	140–145
Ligand H-bonds (max.)	-	6	5	7	4

### Binding free energy calculations

**Fig 14:**
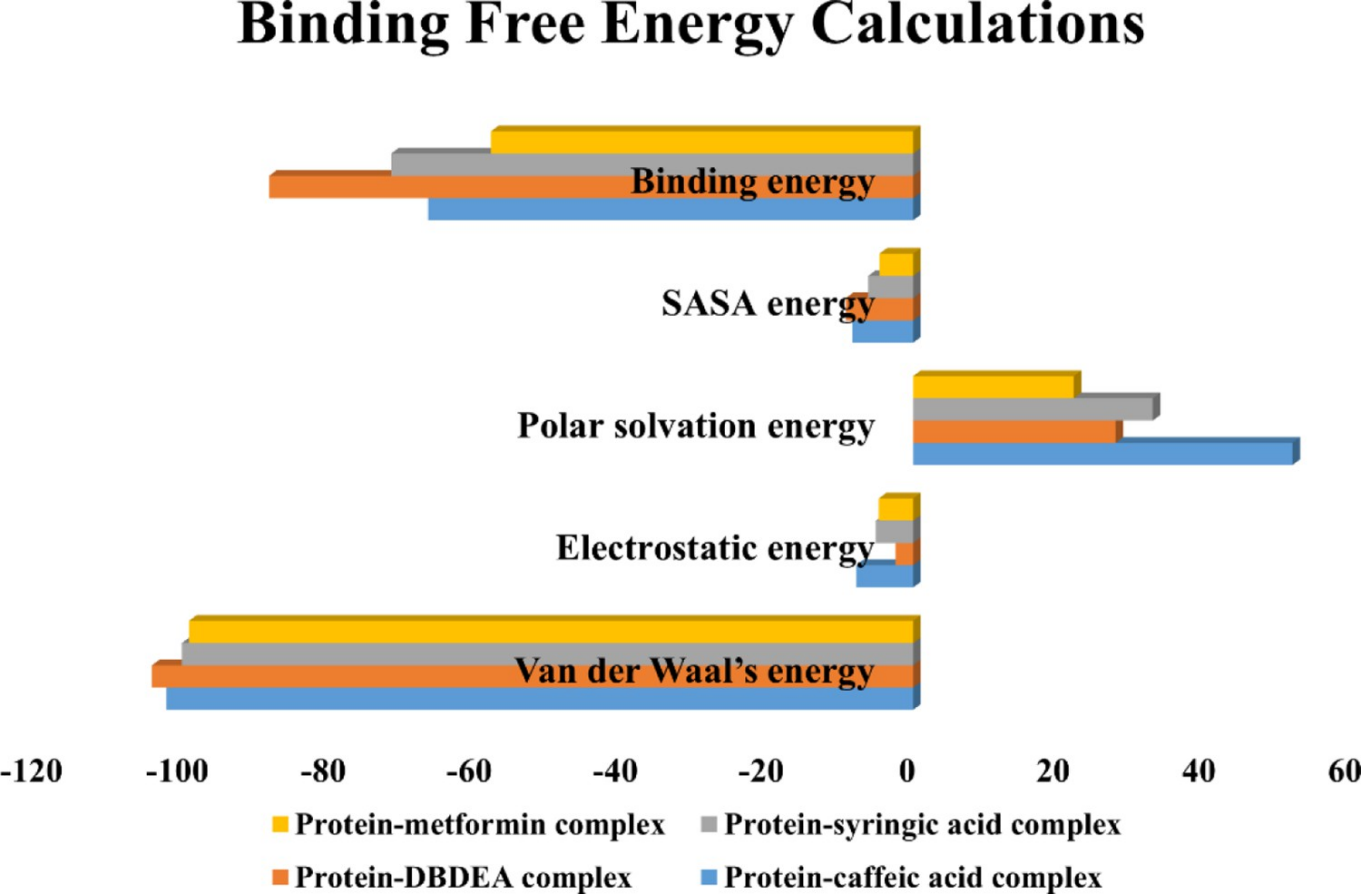
Graphical representation of binding free energies of protein-ligand complexes.

The free energy difference between the completely bound and bonded states is known as binding free energy. The protein’s degree of ligand molecule affinity is determined by an analysis of binding free energy [43]. In this study, based on the free binding energy calculation it can be predicted that van der Waal’s energy and binding energies had a substantial impact on the complex formation. Based on the energy calculation, the predicted results were mostly energetically favorable. According to the predicted result, compound DBDEA bound to human MAPK3 protein (-102.296 kJ/mol) using Van der Waal’s energy showed the highest binding free energy when compared with all the other complexes. The predicted values of energies calculated are summarized in **Table 5** obtained using the MMPBSA technique. Van der Waal’s binding free energy was shown to be the primary contributor to the formation of complexes when compared to the other energies [44] **(Fig 14)**. Further, when compared to protein-control complexes protein-DBDEA complex was found to have higher (more negative) binding free energies, which indicates that it is more stable and requires a higher amount of energy in case of dissociation. These results support the outcomes of both molecular docking and dynamics simulation studies in terms of the overall binding efficiency of the compounds.

**Table 5:** Binding free energy values of JME phytocompounds and metformin complexed with human MAPK3 protein.

Protein-ligand complexes	Types of binding free energies				
	Van der Waal’s energy (kJ/mol)	Electrostatic energy (kJ/mol)	Polar solvation energy (kJ/mol)	SASA energy (kJ/mol)	Binding energy (kJ/mol)
Protein-caffeic acid complex	-102.296	-7.790	51.955	-8.296	-66.427
Protein-DBDEA complex	-104.268	-2.428	27.727	-9.235	-88.203
Protein-syringic acid complex	-100.171	-5.101	32.819	-6.168	-71.514
Protein-metformin complex	-99.189	-4.716	21.991	-4.642	-57.833

### PASS analysis and ADMET profiling of experimental compounds

**Fig 15:**
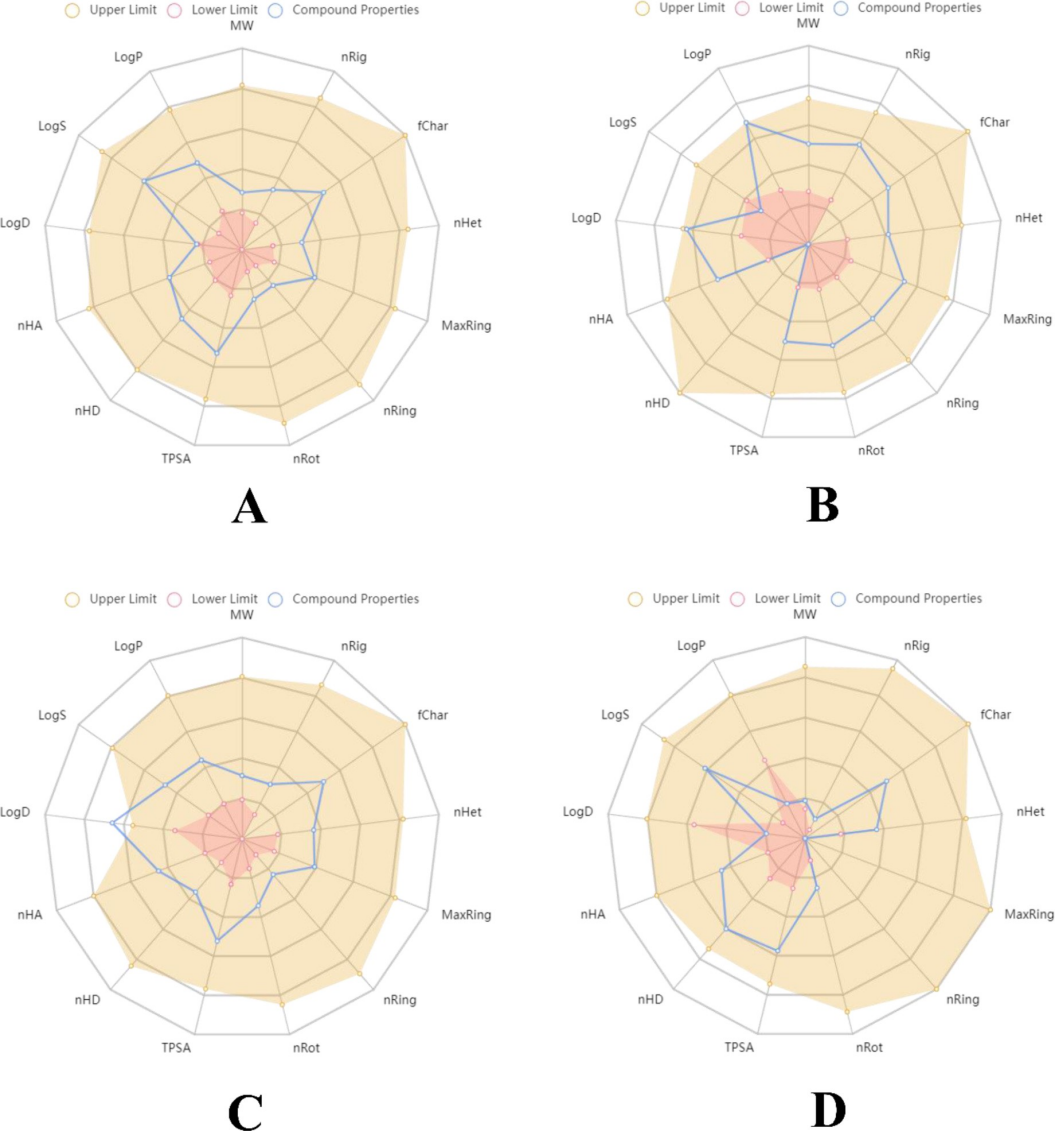
Pharmacokinetic mapping of experimental compounds. A) caffeic acid, B) DBDEA, C) syringic acid, and D) metformin.

During the PASS pharmacological analysis of the experimental compounds used in this study, all the compounds including metformin showed higher “Pa” or probable activity over “Pi” or probable inactivity. It is essential that the chosen compounds should have biological activity, which could be used in the *in vitro* and *in vivo* assays [45]. This shows the compounds could be used to inhibit the MAPK3 protein. Also, the ADMET profiling of the compounds revealed that all the compounds could be treated as safe for oral consumption, without the risk of mutagenicity or allergenicity. The details of the PASS pharmacological activity have been given in **Table 6**, whereas the radar diagrams of the ADMET profiling have been given in **Fig 15**.

**Table 6:** PASS pharmacological activity prediction of experimental compounds.

Compounds	MAPK inhibitor activity	
	Pa	Pi
Caffeic acid	0.242	0.113
DBDEA	0.043	0.015
Syringic acid	0.265	0.08
Metformin	0.551	0.026

## Conclusion

In the treatment of lifestyle-based disorders like obesity and diabetes mellitus (DM), the phytochemical intervention has already been shown to ameliorate problems caused by the sedentary lifestyle. However, several targets in obesity-linked DM need to be diagnosed. Due to the fact that human MAPK3 is a common target for both obesity and DM, and is linked with the insulin signaling pathway, we report the virtual screening of the JME phytocompounds as novel potential inhibitors of human MAPK3 in this work. Using network pharmacology methods, the study methodically pinpoints human MAPK3 as a possible target, which could be inhibited to revitalize insulin levels. This could further bring down the hyperglycemic levels raised in obese individuals. The druglikeness and pharmacokinetic strategy approve a few of the JME phytocompounds as bioactive and orally consumable. Among such compounds, caffeic acid, DBDEA, and syringic acid were considered potential inhibitors of human MAPK3 inhibitors from molecular docking simulation, molecular dynamics simulation, and MMPBSA studies. They bind and interact efficiently with the key residues of the inhibitor binding site of the protein, and stay in the binding pocket without unnecessary fluctuations. These results indicate that the selected compounds could induce biological activity in the macromolecule. These findings led us to the conclusion that selected phytocompounds from JME (caffeic acid, DBDEA, and syringic acid) could be potential inhibitors of human MAPK3. In the future, selected phytocompounds from JME could be taken for *in vitro* and *in vivo* analyses to define their biological activity.

## Supporting information

S1 File Phytochemical profiling of whole green jackfruit flour methanol extract; Phytochemical assessment using HR-LCMS. Phytochemical assessment using GCMS; Phytochemical assessment using RP-HPLC; References.
(DOCX)

S2 File Elucidation of results obtained during phytochemical profiling. (DOCX)

S1 Table HR-LCMS in +electron spray ionization mode study of phytochemical components in methanol extract of green jackfruit flour. (DOCX)

S2 Table HR-LCMS in -electron spray ionization mode study of phytochemical components in methanol extract of green jackfruit flour. (DOCX)

S3 Table GC-MS study of phytochemical components in methanol extract of green jackfruit flour. (DOCX)

S4 Table HPLC study of phenolic components in methanol extract of green jackfruit flour. (DOCX)
