# Supplementary material for: Integrated network pharmacology and molecular modeling approach for the discovery of novel potential MAPK3 inhibitors from whole green jackfruit flour targeting obesity-linked diabetes mellitus
Source: PLoS One. 2023 Jan 30;18(1):e0280847. doi: 10.1371/journal.pone.0280847 (PMC9886246; doi:10.1371/journal.pone.0280847)
Supplement: S1 File — Phytochemical assessment using GCMS; Phytochemical assessment using RP-HPLC; References. (DOCX) [file pone.0280847.s001.docx]

**Phytochemical profiling of whole green jackfruit flour methanol extract**

**Phytochemical assessment using HR-LCMS**

The phytochemical assessment of the extract was done according to the method described by Ramu et al. (2017) [1]. The extract was analyzed at IIT Bombay's advanced analytical instrument facility (SAIF) in Mumbai using high-resolution liquid chromatography and mass spectrometry (HR-LCMS) for the identification of phytoconstituents. Agilent HR-LCMS model-G6550A with 0.01 percent mass resolution was used to prepare chemical fingerprints of the selected medicinal plant extracts. MS was chosen as the acquisition method, with a minimum range of 150 (m/z), a maximum range of 1000 Dalton (m/z), and a scanning rate of one spectrum per second. HIP sampler (100.0 L/min) and binary sampler (0.300 mL/min) were used to maintain liquid chromatography at 40°C. The chromatographic separations were carried out on a column (100 mm × 1.0 mm, particle size 1.8 m; waters) with a 10 μl sample injection and a flush-out factor of 5.0 s. In pump A, the solvent system was 100 percent water, while in pump B, it was 100 percent acetonitrile. The SAIF (IIT Bombay) database, which has over 62,000 patterns, was used to identify components in the extract and analyze them using mass spectrometry HR-LCMS. The unknown component's spectrum was compared to the known component's spectra recorded in the SAIF library.

**Phytochemical assessment using GCMS**

The extract was subjected to GC-MS analysis using the method described by Ramu et al. (2015) [2]. A gas chromatography (Clarus 500 Perkin Elmer, AOC-20i autosampler) interfaced to a mass spectrometer equipped with an Elite-5MS (5 percent Diphenyl/95 percent Dimethyl poly siloxane) fused to a capillary column (30 nm 0.25 nm ID 0.25 m df) was utilized for the sample analysis (2 ml of the extract with 10:1 split ratio). At a constant flow rate of 1ml min-1, helium gas (99.99 percent) was used as the carrier gas. The oven temperature was set to 110°C (isothermal for 2 minutes), then increased by 10°C min-1 to 200°C, then 5°C min-1 to 280°C, before finishing with a 9-minute isothermal at 280°C. Mass spectra were determined at 70 eV with a 0.5-sec scan interval with fragments ranging from 45 to 450 Da. The total time spent running the GC-MS was 36 minutes. By comparing the average peak area to the total areas, the relative quantity (in percentage) of each component was calculated.

**Phytochemical assessment using RP-HPLC**

Bound phenolic compounds from jackfruit flour were extracted according to the method of Bhaskar et al. (2012) [3], with minor modifications. Briefly, the sample (1g) was extracted with ethanol (70%) and hexane to get rid of free sugars, fat, and phenolic compounds. The residual sample was extracted twice with 1M NaOH containing sodium borohydrate (0.5%) under atmospheric nitrogen. The resultant clear supernatants were acidified with 4N HCl till the pH reached 1.5. Released phenolic compounds were identified with RP-HPLC (Prominence-i, LC-2030C Shimadzu). The sample (250 μg/ml) was dissolved in the mobile phase consisting of water, acetic acid, and methanol in an 80:5:15 ratio respectively was filtered and injected to reverse phase C18 column (Shimpak, 4.6 × 250 mm) and eluted with the isocratic mobile phase. The flow rate was maintained at 0.6 ml/min, and an in-built UV detector was used to detect the compounds at 254 nm.

**References**

1. Ramu, R, Shirahatti, PS, Anilakumar KR, Nayakavadi S, Zamee F, Dhananjaya, B.L, et al. Assessment of Nutritional Quality and Global Antioxidant Response of Banana (Musa Sp. CV. Nanjangud Rasa Bale) Pseudostem and Flower. Pharmacognosy Res. **2017**;*9*:S74–S83. 10.4103/pr.pr_67_17

2. Ramu R, Shirahatti PS, Zameer F, Prasad MNN. Investigation of Antihyperglycaemic Activity of Banana (Musa Sp. Var. Nanjangud Rasa Bale) Pseudostem in Normal and Diabetic Rats. J Sci Food Agric**. 2015**;*95*:165–173. https://doi.org/10.1002/jsfa.6698

3. Bhaskar JJ, Chilkunda SMND, Salimath PV. Banana (Musa Sp. Var. Elakki Bale) Flower and Pseudostem: Dietary Fiber and Associated Antioxidant Capacity. J Agric Food Chem. **2012**;60:427–432. https://doi.org/10.1021/jf204539v
