# Supplementary material for: Integrated network pharmacology and molecular modeling approach for the discovery of novel potential MAPK3 inhibitors from whole green jackfruit flour targeting obesity-linked diabetes mellitus
Source: PLoS One. 2023 Jan 30;18(1):e0280847. doi: 10.1371/journal.pone.0280847 (PMC9886246; doi:10.1371/journal.pone.0280847)
Supplement: S2 File — (DOCX) [file pone.0280847.s002.docx]

**Elucidation of results obtained during phytochemical profiling**

The whole green jackfruit flour methanol extract (JME) was found to contain a class of alkaloids, flavonoids, tannins, saponins, and more complex phenolic, phytosterols, oxalates, and phytates, which not only impart color to fruits and vegetables but also possess several physiological functions, including antidiabetic properties. The HR-LCMS, GC-MS, and HPLC results have been given in supplementary tables S1, S2, and S3, respectively. A total of 120 compounds identified in this study were virtually screened using the ADME approach, and selected compounds were taken for network pharmacology-based screening.
