# Supplementary material for: Integrated network pharmacology and molecular modeling approach for the discovery of novel potential MAPK3 inhibitors from whole green jackfruit flour targeting obesity-linked diabetes mellitus
Source: PLoS One. 2023 Jan 30;18(1):e0280847. doi: 10.1371/journal.pone.0280847 (PMC9886246; doi:10.1371/journal.pone.0280847)
Supplement: S1 Table — (DOCX) [file pone.0280847.s003.docx]

**S1 Table**: HR-LCMS in +electron spray ionization mode study of phytochemical components in methanol extract of green jackfruit flour.

| **Sl. No.** | **Compound name** | **Formula** | **Mass** | **RT** | **m/z ratio** | **Difference (ppm)** |
| --- | --- | --- | --- | --- | --- | --- |
| 1 | 6-Hydroxy-2-naphthaleneacetic acid/2-Naphthaleneacetic acid, 6- hydroxyl- | C_12_H_10_O_3_ | 202.0669 | 0.777 | 185.064 | -19.29 |
| 2 | 2-Methylbutyrylglycine | C_7_H_13_NO_3_ | 159.0897 | 1.062 | 160.097 | -0.73 |
| 3 | Hexanoylglycine | C_8_H_15_NO_3_ | 173.1044 | 1.148 | 156.1011 | 4.62 |
| 4 | Leucylproline/1-L-Leucyl-L-Proline | C_11_H_20_N_2_O_3_ | 228.146 | 1.15 | 229.1536 | 5.89 |
| 5 | Galalpha1-3Galbeta1- 4GlcNAcbeta-Sp | C_22_H_38_N_4_O_16_ | 614.2433 | 4.239 | 619.2219 | -24.52 |
| 6 | 3-(4-Hydroxyphenyl)pyruvic acid | C_9_H_8_O_4_ | 180.0421 | 5.136 | 163.0388 | 0.83 |
| 7 | Chlorogenic acid | C_16_H_18_O_9_ | 354.0946 | 5.138 | 355.1018 | 1.30 |
| 8 | Methyl jasmonate | C_13_H_20_O_3_ | 224.1409 | 5.557 | 207.1377 | 1.46 |
| 9 | Lunarine | C_25_H_31_N_3_O_4_ | 437.2305 | 6.504 | 438.2376 | 2.29 |
| 10 | Dihydroquercetin | C_15_H_12_O_7_ | 304.0578 | 7.288 | 287.0544 | 1.75 |
| 11 | Quercetin | C_21_H_20_O_11_ | 448.1 | 7.328 | 449.1073 | 1.31 |
| 12 | 10-nitro,9Z,12Z-octadecadienoic acid | C_18_H_31_NO_4_ | 325.2218 | 8.33 | 308.2213 | 10.88 |
| 13 | Abscisic acid | C_15_H_20_O_4_ | 264.1354 | 8.508 | 247.132 | 3.05 |
| 14 | 9-hydroxy-10E,14Z-octadecadien-12-ynoic acid | C_18_H_28_O_3_ | 292.203 | 9.367 | 275.1998 | 2.83 |
| 15 | 9S-hydroxy-10S,11S-epoxy-12Z,15Z-octadecadienoic acid | C_18_H_30_O_4_ | 310.2139 | 9.586 | 293.2103 | 1.63 |
| 16 | 8-hydroxy-17-octadecene-10,12-diynoic acid | C_18_H_26_O_3_ | 290.1874 | 9.641 | 273.184 | 2.79 |
| 17 | 8-Hydroxy-13Z-octadecene-9,11-diynoic acid | C_18_H_26_O_3_ | 290.1878 | 11.175 | 291.195 | 1.40 |
| 18 | Tetrahydrosappanone A trimethyl ether | C_19_H_22_O_5_ | 330.1462 | 11.726 | 313.1431 | 1.57 |
| 19 | Phytosphingosine | C_18_H_39_NO_3_ | 317.2925 | 11.822 | 318.2997 | 1.62 |
| 20 | CerP(d18:0/16:0) | C_34_H_70_NO_6_P | 619.4811 | 12.035 | 602.4758 | 20.92 |
| 21 | 7-Hydroxyetodolac | C_17_H_21_NO_4_ | 303.1466 | 12.122 | 286.1432 | 1.45 |
| 22 | N-Hexadecyl-L-hydroxyproline | C_21_H_41_NO_3_ | 355.3079 | 12.123 | 356.3152 | 2.18 |
| 23 | Tangeretin | C_20_H_20_O_7_ | 372.1205 | 12.408 | 355.1171 | 0.99 |
| 24 | Rhamnetin | C_16_H_12_O_7_ | 316.0577 | 12.448 | 299.0544 | 1.90 |
| 25 | 1-(6-[5]-ladderane-hexanoyl)- 2-(8-[3]-ladderane-octanyl)-sn-glycerol | C_41_H_64_O_4_ | 617.4675 | 12.581 | 600.4605 | 4879.54 |
| 26 | CerP(d18:1/16:0) | C_34_H_68_NO_6_P | 617.4655 | 12.758 | 600.4603 | 20.99 |
| 27 | Triptonide | C_20_H_22_O_6_ | 358.141 | 12.943 | 341.1377 | 1.71 |
| 28 | 8,13-dihydroxy-9,11- octadecadienoic acid | C_18_H_32_O_4_ | 312.2295 | 13.047 | 295.2262 | 1.88 |
| 29 | Deoxysappanone B 7,3'-dimethyl ether acetate | C_20_H_20_O_6_ | 356.1255 | 13.343 | 339.1222 | 1.33 |
| 30 | 6alpha-Hydroxycastasterone | C_28_H_50_O_5_ | 466.3669 | 13.665 | 471.3453 | -2.25 |
| 31 | 1-Monopalmitin | C_19_H_38_O_4_ | 330.2785 | 13.704 | 353.2676 | -4.49 |
| 32 | (22S)-1alpha,25-dihydroxy-22- methoxy-26,27-dimethyl-23,24-tetradehydro-20- epivitamin D3 / (22S)-1alp | C_30_H_46_O_4_ | 470.3385 | 13.743 | 471.3456 | 2.25 |
| 33 | Cortol | C_21_H_36_O_5_ | 368.2555 | 13.871 | 351.2523 | 1.99 |
| 34 | Linolenoyl lysolecithin | C_26_H_48_NO_7_P | 517.3163 | 13.991 | 518.3237 | 1.02 |
| 35 | Tiamulin | C_28_H_47_NO_4_S | 493.3169 | 14.403 | 494.3229 | 11.54 |
| 36 | Typhasterol | C_28_H_48_O_4_ | 448.3562 | 14.525 | 453.3348 | -2.13 |
| 37 | 6-Deoxotyphasterol | C_28_H_50_O_3_ | 434.3769 | 14.771 | 439.3549 | -2.09 |
| 38 | GPEtn(10:0/11:0)[U] | C_26_H_52_NO_8_P | 537.3422 | 14.978 | 520.3391 | 1.56 |
| 39 | 13S-hydroxy-9E,11Z-octadecadienoic acid | C_18_H_32_O_3_ | 296.2348 | 15.019 | 279.2316 | 1.32 |
| 40 | 6-Deoxocastasterone | C_28_H_50_O_4_ | 450.372 | 15.270 | 455.3507 | -2.39 |
| 41 | 6-Deoxoteasterone | C_28_H_50_O_3_ | 434.3761 | 15.313 | 457.3659 | -0.35 |
| 42 | 3alpha,12alpha,25-trihydroxy-5beta-cholestan-7-one | C_28_H_50_O_4_ | 450.3715 | 15.396 | 473.3609 | -1.22 |
| 43 | (Z)-2-tetracos-15- enamidoethanesulfonic acid/N-Nervonoyltaurine | C_26_H_51_NO_4_S | 473.3525 | 15.524 | 496.3387 | 3 |
| 44 | 9S,10-epoxy-10,12Z-octadecadienoic acid | C_18_H_30_O_3_ | 294.2195 | 15.788 | 277.216 | 0.06 |
| 45 | Heptylene-bis(tacrine) | C_33_H_40_N_4_ | 492.3281 | 15.975 | 493.3308 | -5.6 |
| 46 | ZK 168281 | C_32_H_46_O_5_ | 508.3299 | 15.994 | 491.3181 | 3943.52 |
| 47 | 3-Dehydro-6-deoxoteasterone | C_28_H_48_O_3_ | 432.3614 | 16.351 | 437.3402 | -2.52 |
| 48 | N-Acetylsphingosine | C_20_H_39_NO_3_ | 341.2922 | 16.569 | 324.2889 | 2.39 |
| 49 | EB 1129 | C_30_H_46_O_3_ | 454.3439 | 18.172 | 437.3406 | 1.84 |
| 50 | 5beta-Cholestane-3alpha,7alpha,12alpha,25,26-pentol | C_27_H_48_O_5_ | 452.3449 | 18.265 | 453.3521 | 11.74 |
| 51 | 19-oxo-22Z-octacosenoic acid | C_28_H_52_O_3_ | 436.3926 | 19.087 | 441.3712 | -2.24 |
