# Supplementary material for: Integrated network pharmacology and molecular modeling approach for the discovery of novel potential MAPK3 inhibitors from whole green jackfruit flour targeting obesity-linked diabetes mellitus
Source: PLoS One. 2023 Jan 30;18(1):e0280847. doi: 10.1371/journal.pone.0280847 (PMC9886246; doi:10.1371/journal.pone.0280847)
Supplement: S2 Table — (DOCX) [file pone.0280847.s004.docx]

**S2 Table:** HR-LCMS in -electron spray ionization mode study of phytochemical components in methanol extract of green jackfruit flour

| **Sl. No.** | **Compound name** | **Formula** | **Mass** | **RT** | **m/z ratio** | **Difference (ppm)** |
| --- | --- | --- | --- | --- | --- | --- |
| 1 | Quinic acid | C_7_H_12_O_6_ | 192.0631 | 1.111 | 191.0558 | 1.44 |
| 2 | Sericetin diacetate | C_29_H_28_O_7_ | 488.1735 | 1.112 | 533.1718 | 20.54 |
| 3 | 5'-Phosphoribosylglycinamide (GAR) | C_7_H_15_N_2_O_8_P | 286.0531 | 8.276 | 285.0458 | 12.09 |
| 4 | 2,3-dinor-PGE1 | C_18_H_30_O_5_ | 326.2066 | 9.003 | 325.1993 | 8.48 |
| 5 | 11-hydroperoxy-12,13-epoxy-9-octadecenoic acid | C_18_H_32_O_5_ | 328.2234 | 9.063 | 327.2161 | 4.65 |
| 6 | Isobutylglycine | C_11_H_16_N_4_O_4_ | 268.1158 | 9.178 | 327.1295 | 5.13 |
| 7 | 9S,10S,11R-trihydroxy-12Z-octadecenoic acid | C_18_H_34_O_5_ | 330.2393 | 9.555 | 329.232 | 4.12 |
| 8 | 1-(5-Ketohexyl)-3- methylxanthine | C_12_H_16_N_4_O_3_ | 264.1207 | 11.108 | 309.1188 | 5.85 |
| 9 | Tyrphostin B44 (- | C_18_H_16_N_2_O_3_ | 308.112 | 12.048 | 353.1103 | 13.35 |
| 10 | p,gamma-Dihydroxyphenylbutazone | C_19_H_20_N_2_O_4_ | 340.1378 | 12.649 | 339.1305 | 13.24 |
| 11 | Isovalerylglucuronide | C_11_H_18_O_8_ | 278.105 | 12.682 | 337.1138 | 0.57 |
| 12 | Caloxanthin sulfate | C_40_H_56_O_6_S | 664.3663 | 14.164 | 723.3799 | 20.26 |
| 13 | 13R-hydroxy-9E,11Z-octadecadienoic acid | C_18_H_32_O_3_ | 296.2353 | 14.875 | 295.228 | -0.41 |
| 14 | 9,12,14-octadecatrienoic acid | C_18_H_30_O_2_ | 278.2245 | 18.02 | 277.2175 | 0.31 |
| 15 | 4-hydroxy palmitic acid | C_16_H_32_O_3_ | 272.241 | 18.549 | 271.2337 | -21.51 |
| 16 | 13E,17-octadecadienoic acid | C_18_H_32_O_2_ | 280.2394 | 19.112 | 279.2324 | 2.82 |
| 17 | 2-propyl-tridecanoic acid | C_16_H_32_O_2_ | 256.2391 | 20.103 | 255.2319 | 4.34 |
| 18 | 5-octadecylenic acid | C_18_H_34_O_2_ | 282.2546 | 20.269 | 281.2473 | 4.61 |
| 19 | (+)-3-hydroxy behenic | C_22_H_44_O_3_ | 356.3272 | 20.438 | 355.3199 | 5.31 |
| 20 | Didesmethylimipramine | C_17_H_20_N_2_ | 252.1607 | 21.711 | 297.1592 | 7.54 |
| 21 | Praziquantel | C_19_H_24_N_2_O_2_ | 312.182 | 22.248 | 311.1748 | 5.81 |
| 22 | Quinine | C_20_H_24_N_2_O_2_ | 324.1817 | 22.656 | 323.1745 | 6.33 |
| 23 | Ajmaline | C_20_H_26_N_2_O_2_ | 326.198 | 22.669 | 325.1909 | 4.53 |
| 24 | GPGro(18:1(9Z)/0:0)[U] | C_24_H_47_O_9_P | 510.2957 | 22.924 | 555.2942 | 0.18 |
| 25 | Dihydrocelastryl diacetate | C_33_H_44_O_6_ | 536.3115 | 23.228 | 581.31 | 4.24 |
| 26 | Trimipramine | C_20_H_26_N_2_ | 294.2082 | 23.405 | 339.2066 | 4.61 |
