# Supplementary material for: Integrated network pharmacology and molecular modeling approach for the discovery of novel potential MAPK3 inhibitors from whole green jackfruit flour targeting obesity-linked diabetes mellitus
Source: PLoS One. 2023 Jan 30;18(1):e0280847. doi: 10.1371/journal.pone.0280847 (PMC9886246; doi:10.1371/journal.pone.0280847)
Supplement: S3 Table — (DOCX) [file pone.0280847.s005.docx]

**S3 Table:** GC-MS study of phytochemical components in methanol extract of green jackfruit flour

| **Sl. No.** | **Name of the compound** | **Molecular formula** | **Molecular weight (g/mol)** | **Probability match (%)** |
| --- | --- | --- | --- | --- |
| 1 | (1-Cyclopropylethyl)methylamine | C_6_H_13_N | 99.17. | 37.0 |
| 2 | 1,2-15,16-Diepoxyhexadecane | C_16_H_30_O_2_ | 254.41 | 9.01 |
| 3 | 1,3-Dithiolane, 2-(28-norurs-12-en-17-yl)- | C_32_H_52_S_2_ | 500.9 | 5.29 |
| 4 | 1,6,10,14,18,22-Tetracosahexaen-3-ol, 2,6,10,15,19,23-hexamethyl-, (all-E)- | C_30_H_50_O | 426.7 | 12.4 |
| 5 | 1b,5,5,6a-Tetramethyl-octahydro-1-oxa-cyclopropa[a]inden-6-one | C_13_H_20_O_2_ | 208.30 | 6.04 |
| 6 | 2,2-Dimethyl-6-methylene-1-[3,5-dihydroxy-1-pentenyl]cyclohexan-1-perhydrol | C_14_H_24_O_4_ | 256.34 | 7.79 |
| 7 | 2,5-Dimethyl-4-hydroxy-3(2H)-furanone | C_6_H_8_O_3_ | 128.13 | 44.7 |
| 8 | 2-Cyclopentene-1-undecanoic acid | C_16_H_28_O_2_ | 252.39 | 11.8 |
| 9 | 2-furancarboxaldehyde 5-(hydroxymethyl)- | C_6_H_6_O_3_ | 126.11 | 15.3 |
| 10 | 3-amino-2-oxazolidine | C_3_H_6_N_2_O_2_ | 102.09 | 11.4 |
| 11 | 4H-Pyran-4-one, 2,3-dihydro-3,5-dihydroxy-6-methyl- | C_6_H_8_O_4_ | 144.12 | 83.9 |
| 12 | 9,12,15-Octadecatrienoic acid, 2-[(trimethylsilyl)oxy]-1-[[(trimethylsilyl)oxy]methyl]ethyl ester, (Z,Z,Z)- | C_27_H_52_O_4_Si_2_ | 496.9 | 33.8 |
| 13 | 9,12-Octadecadienoic acid (Z,Z)-, phenylmethyl ester | C_25_H_38_O_2_ | 370.56 | 46.1 |
| 14 | 9,12-Octadecanoic acid | C_18_H_32_O_2_ | 280.4 | 11.4 |
| 15 | 9,19-Cycloergost-24(28)-en-3-ol, 4,14-dimethyl-, acetate, (3.beta.,4.alpha.,5.alpha.)- | C_32_H_52_O_2_ | 468.75 | 10.8 |
| 16 | 9,19-Cyclolanost-24-en-3-ol, acetate, (3beta)- | C_32_H_52_O_2_ | 468.8 | 15.5 |
| 17 | 10-Undecenoic acid, methyl ester | C_12_H_22_O | 182.13 | 11.2 |
| 18 | 17-Octadecynoic acid | C_18_H_32_O_2_ | 280.4 | 14.2 |
| 19 | Cyclopentaneundecanoic acid | C_16_H_30_O_2_ | 254.41 | 8.70 |
| 20 | Cyclopropanecarboxamide, N-(2-methyl-2-heptyl) | C_12_H_23_N_O_ | 197.32 | 15.9 |
| 21 | Cyclopropyl carbinol | C_4_H_8_O | 72.1057 | 14.2 |
| 22 | C(14a)-Homo-27-norgammacer-13-en-21beta-ol, 3alpha-methoxy- | C_31_H_50_O2 | 456.7 | 7.87 |
| 23 | cis-(Z)-alpha-Bisabolene epoxide | C_15_H_24_ | 220.35 | 8.59 |
| 24 | Methyl 4-O-acetyl-2,3,6-tri-O-ethyl-alpha-D-galactopyranoside | C_15_H_28_O_7_ | 320.14 | 15.1 |
| 25 | n-hexadecanoic acid | C_16_H_32_O_2_ | 256.42 | 65.8 |
| 26 | Oleic acid | C_18_H_34_ | 282.5 | 53.1 |
| 27 | Squalene | C_30_H_50_ | 410.7 | 12.9 |
| 28 | Stigmasta-4,6,22-trien-3beta-ol | C_29_H_46_O | 410.7 | 11.6 |
| 29 | Tridecanoic acid, methyl ester | C_14_H_28_O_2_ | 228.37 | 20.3 |
| 30 | Urs-12-en-28-ol | C_30_H_50_O | 426.7 | 8.71 |
