# Supplementary material for: Integrated network pharmacology and molecular modeling approach for the discovery of novel potential MAPK3 inhibitors from whole green jackfruit flour targeting obesity-linked diabetes mellitus
Source: PLoS One. 2023 Jan 30;18(1):e0280847. doi: 10.1371/journal.pone.0280847 (PMC9886246; doi:10.1371/journal.pone.0280847)
Supplement: S4 Table — (DOCX) [file pone.0280847.s006.docx]

**S4 Table:** HPLC study of phenolic components in methanol extract of green jackfruit flour

| **Sl. No.** | **Name** | **Molecular formula** | **Ret. time** | **Area** | **Height** |
| --- | --- | --- | --- | --- | --- |
| 1 | Ascorbic acid | C_6_H_8_O_6_ | 4.223 | 294491 | 35608 |
| 2 | Gallic acid | C_7_H_6_O_5_ | 5.712 | 96769 | 16767 |
| 3 | Catechin | C_15_H_14_O_6_ | 11.520 | 73497 | 7891 |
| 4 | Methyl gallate | C_8_H_8_O_5_ | 12.436 | 30776 | 3117 |
| 5 | Caffeic acid | C_9_H_8_O_4_ | 14.586 | 21652 | 2539 |
| 6 | Syringic acid | C_9_H_10_O_5_ | 15.033 | 14842 | 2119 |
| 7 | Rutin | C_27_H_30_O_16_ | 18.396 | 11618 | 1976 |
| 8 | p-Coumaric acid | C_9_H_8_O_3_ | 19.068 | 15034 | 2077 |
| 9 | Sinapic acid | C_11_H_12_O_5_ | 19.851 | 38537 | 6079 |
| 10 | Ferulic acid | C_10_H_10_O_4_ | 20.996 | 55995 | 6370 |
| 11 | Quercetin | C_15_H_10_O_7_ | 28.368 | 11515 | 1267 |
| 12 | Apigenin | C_15_H_10_O_5_ | 32.265 | 96531 | 8183 |
| 13 | Kaempferol | C_15_H_10_O_6_ | 34.993 | 107714 | 10321 |
